# Stinging Trichome Density and Morphology of Three Nettle Species Reflect Mountain Gorillas' Feeding Behavior

**DOI:** 10.1002/ece3.71765

**Published:** 2025-07-22

**Authors:** Alphonse Nyandwi, Winnie Eckardt, Elias Bizuru, Myriam Mujawamariya, Melanie L. DeVore

**Affiliations:** ^1^ Department of Biology University of Rwanda Kigali Rwanda; ^2^ Dian Fossey Gorilla Fund Musanze Rwanda; ^3^ Department of Biological & Environmental Sciences Georgia College and State University Milledgeville Georgia USA

**Keywords:** mountain gorillas, nettle, plant defense, trichome density, trichomes

## Abstract

Plants have developed defense mechanisms against herbivory, including stinging trichomes. Unlike smaller trichomes, which deter insects, the larger, biomineralized, stinging trichomes in Urticaceae defend plants from mammals. The trichome tip breaks off, pierces the skin, and injects irritants, causing an immediate sensation of pain. The herbivore will cease consuming the plant. Some herbivores process and consume nettles. Volcanoes National Park (VNP) in Rwanda provides habitat for several large folivores, including the endangered mountain gorilla ( *Gorilla beringei beringei* ). VNP mountain gorillas feed on stinging nettle species, including *Laportea alatipes*, *Urtica massaica*, and *Girardinia bullosa*. We investigated the link between the importance (proportion) of these stinging nettles in the diet of gorilla groups ranging in the southwest of VNP and their level of defense through assessing trichome density, length, and glandular base length from each plant organ of 15 specimens per nettle species, which were photographed with a scale ruler under a digital microscope. We also videotaped 12 mountain gorillas consuming *L. alatipes* to examine adapted feeding techniques to cope with stinging trichomes. We found that *L. alatipes*, which is consumed most frequently of the three study nettles, had a significantly higher stinging trichome density compared to *U. massaica* and *G. bullosa*. However, the length of stinging trichomes and glandular bases containing irritating secretions were significantly smaller in *L. alatipes* and *U. massaica* than in *G. bullosa*, from which gorillas almost exclusively avoid consuming aboveground organs covered with long trichomes. This suggests that larger stinging trichomes and secretory glandular bases offer a more effective defense against mountain gorillas than increased trichome density. The trichome density of *L. alatipes* tended to be higher on top leaves and stem sections, which are consumed by gorillas more frequently compared to lower parts. Plants have evolved in an intricate way to adapt to herbivores' attacks.

## Graphical abstract

Our study analyzed the density and morphology of stinging trichomes in three nettle species and related these characteristics to the feeding behaviors of mountain gorillas. Despite their physical characteristics and ability to sting, mountain gorillas rely on these plants as their staple food source.

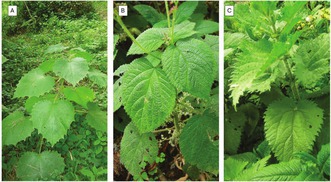

## 1 Introduction

Species interactions have always been the “fabric” that holds communities together (Hagen et al. 2012; Jordano 2016). Because herbivores depend on plants for food, there is an exceptional coevolutionary relationship between plants and these primary consumers (Baraza et al. 2007; Burkepile and Parker 2017). Plants are challenged with having to adapt to both unpredictable climatic events and the pressure from heterotrophic organisms (Mithöfer and Boland 2012; Orrock et al. 2015). Since plants are sessile organisms and thus unable to immediately retreat, they have adapted their anatomy and physiology in concert with secondary compounds (Yang et al. 2018) and employ multiple defensive strategies to deter herbivores (Pullin and Gilbert 1989; Hanley et al. 2007; Yang et al. 2018; War et al. 2018). For example, plants evolved a diversity of effective chemical, mechanical, and structural defenses (Ågren and Schemske 1993; Coley and Barone 1996; Hanley et al. 2007). Some families, such as Urticaceae, Euphorbiaceae, Loasaceae, and Hydrophyllaceae, have evolved stinging trichomes (Thurston 1969; Thurston and Lersten 1969; Thurston 1974; Ensikat et al. 2021). The Urticaceae family has long, since the early Eocene period, developed stinging trichomes to protect themselves from large herbivores (DeVore et al. 2020).

Stinging trichomes combine both mechanical and chemical defense (Ensikat et al. 2021) and are classified as glandular and nonglandular, with the former providing an additional chemical defense (Schilmiller et al. 2008; Mustafa et al. 2018). Glandular trichomes have the peculiarity of synthesizing, storing, and, in the case of Urticaceae, secreting large quantities of histamine and acetylcholine (Grauso et al. 2020). Contrary to this, nonglandular trichomes did not evolve a secretory mechanism (Karabourniotis et al. 2020). Stinging trichomes of the tribe Urticae (Urticaceae) are composed of hollow, mineralized cells and are a combination of cells responsible for producing the mineralized sharp trichome that acts as a syringe when compressed by a mechanical force, injecting the substances into the herbivore. The mineralized tip easily penetrates and injects into the skin an irritant liquid (secondary metabolites) released from their glands that causes pain (Fu et al. 2007; Schilmiller et al. 2008). Although stinging trichomes cannot completely prevent herbivory (Dalin et al. 2008), they do serve to reduce the number of herbivore attacks, because feeding often ceases immediately, preventing further damage to the plant when the herbivore senses pain (Pollard and Briggs 1984; Pullin and Gilbert 1989; Kato et al. 2008; Shikata et al. 2013). In contrast, invertebrates are less affected by stinging trichomes and can feed around trichomes (Iwamoto et al. 2014).

Previous studies attempted to analyze the mammal herbivore‐nettle interaction by either herbivore simulation or actual observations in the wild. In assessing the results of these investigations, it is important to remember that stinging trichomes are highly specialized epidermal structures and a derived evolutionary character (Mustafa et al. 2018). Stinging trichomes induce a herbivore to immediately sense pain and cease consuming the plant (Mustafa et al. 2018; Ensikat et al. 2021). Results from previous studies found that plants that have been exposed long‐term to intense damage or grazing by large mammals have a substantially increased trichome density compared to those from less browsed plants (Pollard and Briggs 1984; Kato et al. 2008; Iwamoto et al. 2014). For instance, the stinging nettle *Urtica thurnbergiana* is exposed to intense deer browsing in Nara Park, Japan, and is equipped with a higher trichome density than its counterparts located in areas with low or no deer browsing (Iwamoto et al. 2014). Moreover, an herbivore simulation through leaf cutting on *Urtica dioica* also revealed a significant increase in trichome density in regrowing leaves (Pullin and Gilbert 1989; Mutikainen and Walls 1995). It is thus suggested that, subsequent to mechanical damage, nettle exhibits ecological strategies to allocate resources for defense in regrowing organs to deter further herbivory (Pullin and Gilbert 1989; Fordyce and Agrawal 2001; Traw and Dawson 2002).

Gorillas (*Gorilla*) incorporate herbaceous foods into their diet at varying amounts, with mountain gorillas ( *Gorilla beringei beringei* ), which are found in two separated populations, the Sarambwe‐Bwindi Ecosystem and the Virunga massif, relying most on terrestrial herbs and least on fruits among the four gorilla subspecies (Harcourt and Stewart 2007). The Virunga mountain gorillas live at an extreme compared to all other gorilla populations, ranging at the highest elevational range where fruiting trees are lacking and terrestrial herbaceous understory is very dense (Watts 1984).

The Volcanoes National Park (VNP), which is the Rwandan part of the Virunga massif, consists of steep slopes of tropical montane rainforest that harbors a variety of other large herbivores, including the African elephant ( *Loxodonta africana* ), African buffalo ( *Syncerus caffer* ), bushbuck ( *Tragelaphus scriptus* ), and black‐fronted duiker ( *Cephalophus nigrifrons* ) that have a paramount effect on vegetation (Plumptre 1996; Owiunji et al. 2005). The flora within the VNP includes stinging nettle plants from the Urticaceae family known to be capable of responding to high levels of perturbation by large mammalian herbivores (Watts 1984). Plumptre (1991) estimated based on cuticle fragments in fecal samples, collected in 1988 and 1989, that 14% of the gorilla plant diet was *Laportea*, while this nettle species was also identified in samples from elephant (11.2%), bushbuck (9.7%), and duiker (0.1%) but not buffalo. Unpublished results from a survey targeting the same herbivores conducted by Dushimirimana in 2018 (2019, unpublished) indicated the presence of *Laportea* only in elephant and gorilla fecal materials. Neither study found evidence of other species of nettles in these mammals' diet. This kind of ecosystem marks an ideal model to study the coevolved species and shed light on defensive strategies developed by stinging nettles.

Mountain gorillas in the VNP mainly feed on barks, roots, stems, flowers, leaves, and very few fruits of plants that are available all year round (Watts 1984; Plumptre 1991). Bamboo shoot is one of the few seasonally available gorilla foods, coinciding with the two rainy seasons (Watts 1984). Although VNP mountain gorillas consume parts of at least 200 plant species, 80% of their diet is made up of only ~4–13 food plant species (herein called key foods) with most other food plant species accounting for less than 0.1% of feeding observations (Grueter et al. 2013; Ihimbazwe et al. 2025; Watts 1984). The composition of key foods can vary substantially among groups depending on the location of their home range within the VNP (Ihimbazwe et al. 2025), which is characterized by a mosaic of habitat types that differ in their availability of food resources (Watts 1984). Mountain gorillas in the VNP appear to not be completely deterred by stinging trichomes (McNeilage 2001; Tennie et al. 2008). Nettles of Urticaceae are a key food for mountain gorillas in some VNP areas, such as for groups ranging on the slopes and in the saddle of the two volcanoes, Bisoke and Karisimbi, in the so‐called Karisoke research area (Grueter et al. 2013; Ihimbazwe et al. 2025; McNeilage 2001; Watts 1984). These groups consume specific stinging nettles more frequently than groups ranging in the eastern part of the VNP (Ihimbazwe et al. 2025), although nettles make up an overall small proportion of their diet (Grueter et al. 2013). Among these stinging nettles, *Laportea alatipes* ranks among the five most important gorilla key food plants, covering 2.9%–6.2% of feeding observations in the Karisoke research area (Ihimbazwe et al. 2025; Grueter et al. 2013). Other nettles consumed by mountain gorillas in the study area are categorized as stinging: *Urtica massaica* (0.1%), *Girardinia bullosa* (< 0.1%), and nonstinging: *Drogetia iners* (2%), *Urera hypselodendron* (< 0.1%) (Vedder 1984; Watts 1984; Plumptre 1991; McNeilage 2001; Grueter et al. 2013).

**FIGURE 1:**
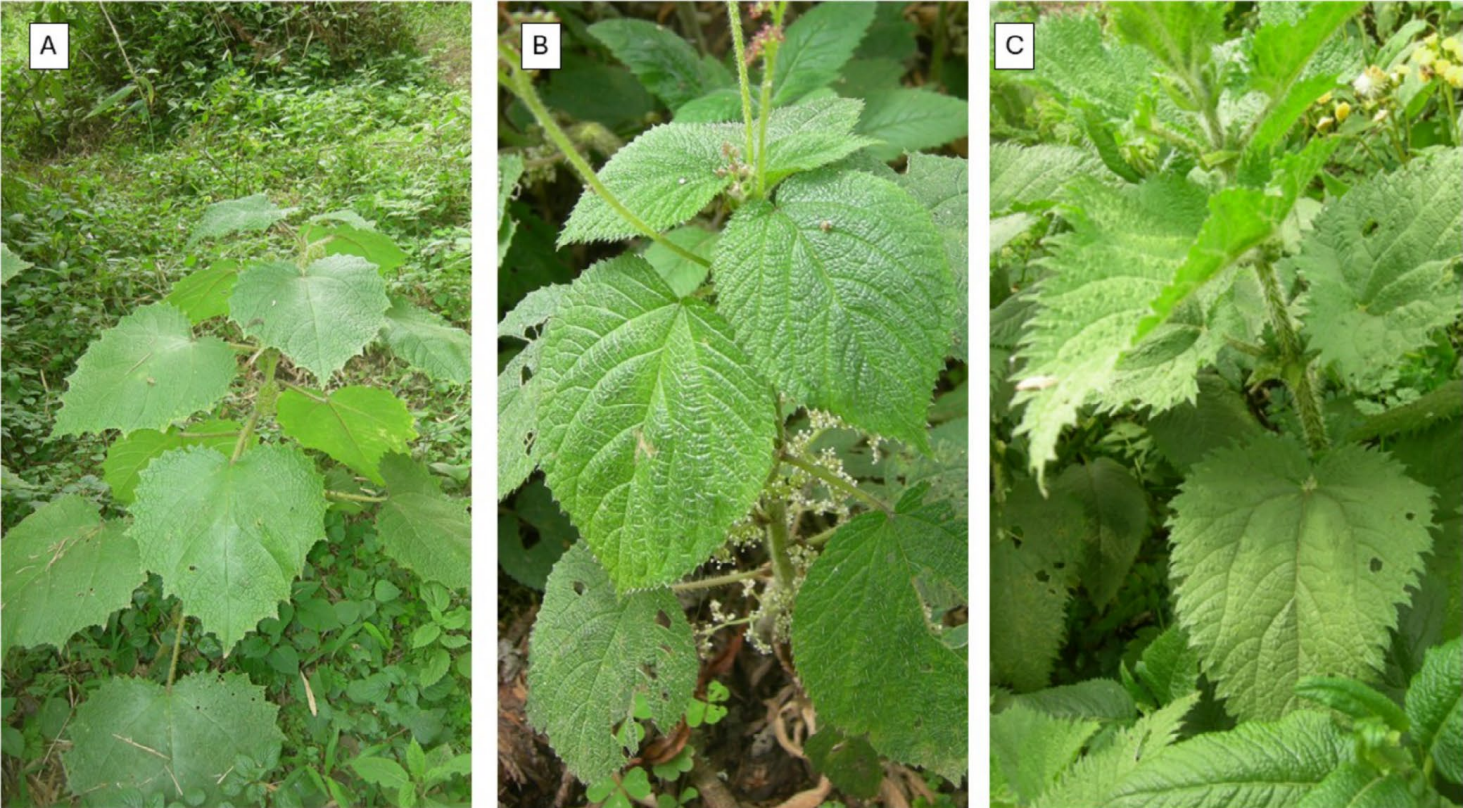
The three nettle species included in this study: *Girardinia bullosa* (A), *Laportea alatipes* (B), and *Urtica massaica* (C).

Despite morphological traits and the stinging ability, mountain gorillas harvest *L. alatipe*s through skillful sequential feeding techniques, from plant acquisition to food item ingestion (Byrne and Byrne 1993). With this regard, Byrne and Byrne (1993) studied and described these techniques as follows: pulling and holding closer to the nettle stem; stripping leaves up the stem; detaching and dropping off petioles and twisting using the second hand; folding leaf blades over the thumb; pulling out the thumb while regrasping the leaf bundle; and finally moving the parcel into the mouth. However, the stinging trichome densities and morphology of the various stinging nettle species and their plant parts consumed by mountain gorillas in the VNP in response to observed feeding techniques have not yet been studied, compared, and linked to feeding frequencies. This study aimed to address these knowledge gaps by measuring the length of stinging trichomes and the glandular trichome base and calculating trichome density by incorporating the three most frequently consumed stinging nettle species by mountain gorillas ranging in the Karisoke research area, namely *L. alatipes*, *U. massaica*, and *G. bullosa* (Figure 1A–C). Mountain gorillas in the Karisoke research area consume the leaves, flowers, stem, and root of *L. alatipes* and *U. massaica*, whereas they only target the root of *G. bullosa* (McNeilage 2001; Watts 1984). *L. alatipes* and *U. massaica* are known to be nutritious foods and provide medicinal benefits (Devkota et al. 2022; Mahlangeni et al. 2020). We then compared trichome density and length as well as glandular trichome base length measures of these food plant parts between species and within nettle species, except for roots, which have no trichomes, to test whether there is a relationship with their importance in mountain gorilla diets (% of total diet). For between‐species comparison, we hypothesized that *L. alatipes*, which is consumed most frequently, would have the highest trichome density of the three nettle species, while the less consumed *G. bullosa* has adapted longer trichomes and glandular base length for effective defense.

Finally, we investigated whether trichome density, trichome length, and glandular trichome base length on *L. alatipes* leaves and stems reflect adaptation of this key food plant in response to predation by mountain gorillas, using feeding events on this species recorded on videos in addition to existing feeding technique descriptions (Byrne and Byrne 1993; Byrne et al. 2011). We expect the highest trichome density and length and the longest glandular trichome base on plant organs and the locations of organs that are manipulated during gathering and harvesting. While Byrne and Byrne (1993) described in detail the harvesting and processing techniques used by mountain gorillas when feeding on *L. alatipes*, they did not provide information about which stem sections (upper, middle, and lower) and leaves along the stem (upper, middle, and lower) were manipulated and consumed and which leaf surface (upper or lower) was predominantly touched and damaged by gorillas during leaf harvesting. We aim to fill this gap and link such information with data on trichome density and length, as well as glandular trichome base length. Based on the described feeding techniques used by mountain gorillas when feeding on *L. alatipes* (Byrne and Byrne 1993; Byrne 1999), we expect that the top stem sections and leaves, as well as the lower leaf surfaces, would have higher trichome densities and shorter trichome length and glandular trichome bases than the middle and bottom stem sections and leaves on those sections, as well as the upper leaf surface, reflecting the response to damage and adapted feeding techniques while stripping the leaves off the stem. The present study will advance our knowledge of the coevolution of mountain gorillas and three of their nettle food plant species in the Virunga massif.

## 2 Materials and Methods

### 2.1 Study Area and Species

The study was carried out in the Volcanoes National Park (VNP) in Rwanda 1°20′–1°30′ S, 29°20′–29°40′ longitude E. VNP is part of the Virunga massif that forms a contiguous tropical montane rainforest with two other protected areas, the Virunga National Park in the Democratic Republic of the Congo and the Mgahinga Gorilla National Park in Uganda (Kalpers et al. 2003). VNP covers an elevation range from 2300 to 4500 m and eight distinct vegetation types (Plumptre 1991; McNeilage 2001; Grueter et al. 2013). Annual rainfall in the region is distributed bimodally, resulting in two rainy seasons (March–May, September–November) and two dry seasons (June–August, December–February), with an average of approximately 2000 mm (Plumptre 1991; Karger et al. 2017). The temperature is mild year‐round and changes from low to high altitude (Mehta and Katee 2005).

Our study area was restricted to the southwest slopes of Mount Bisoke, where some of the highest gorilla densities are found within the Virunga massif (Granjon et al. 2020; Gray et al. 2010, 2013). Three nettle species with stinging trichomes consumed by mountain gorillas in this part of the VNP occur (Watts 1984) and were included in this study, namely *Urtica massaica* Mildbr, *Girardinia bullosa* (Hochst.ex Steud.) Wedd., and *Laportea alatipes* Hook.f. (Friis 1989). African elephants that feed on *L. alatipes* (Plumptre 1991; Dushimirimana 2019, unpublished) have been absent in this study area for over two decades (see Owiunji et al. 2005; Twahirwa et al. 2023). In addition, the most recent study (Dushimirimana 2019, unpublished) on the diet of buffalo, bushbuck, and duikers in the study area suggests that they either do not consume the three study nettles or only consume them at negligible proportions undetectable by fecal analysis. We therefore assume the manipulation and removal of nettle leaves and stems by gorillas is the likely driver for trichome morphology and density. Thus, we focused our investigation on gorilla feeding behavior.

The study nettles are described as herbs characterized by epidermal cells covered in numerous short, nonstinging, and stinging trichomes that are sharply hooked and easily penetrate the skin (Friis 1989; A. Nyandwi, personal observation). *Urtica* is a dioecious perennial herb erecting up to 2 m tall, with a stem that is little branched and quadrangular and with an acuminate apex. The opposite and lanceolate‐shaped leaves have serrated margins and secondary veins that alternate. *Girardinia* is an annual monocarpic herb with a hollow stem that is unbranched or little branched near the top, somewhat lignified, and grows up to 3 m tall. Leaves and secondary veins are alternated. The leaf margins are double‐serrated. *Laportea* is a monoecious, robust, perennial herb up to 1.5 m tall with a soft, erect, and little‐branched stem that is usually lignified at the base. The leaves alternate, are shaped broadly lanceolate to ovate, and have a serrated margin and axially inflorescence (Friis 1989; A. Nyandwi, personal observation).

### 2.2 Field Sample Collection

Upon research approval from the Rwanda Development Board, we collected nettle specimens supported by the Dian Fossey Gorilla Fund's (Fossey Fund) field staff between June and July 2016. For each of the three study nettle species, we selected three sampling sites in 200‐m elevational intervals (at approximately 2600, 2800, and 3000 m) based on the experience of the field staff, where each of the nettle plants occurs in high densities. At each sampling site, we established a 5 m × 5 m plot in which we selected five individuals, resulting in a total of 15 specimens for each study nettle species across all sampling sites. To maintain data uniformity, we selected plants of approximately 1.3 m in height to avoid specimens that lost lower leaves due to senescence.

After selecting an individual plant at a site, we harvested the specimen following the method used by Shikata et al. (2013) and selected three leaves, with their respective petioles, of similar surface size and shape from different nodes. We sampled an undamaged leaf from the bottom, middle, and top of each stem. In addition, we collected the flower stalk and three portions of the stem, approximately 15 cm in length, one from each of the bottom, middle, and top stem parts. To keep the collected samples fresh and to avoid any damage, we transported the samples in plastic envelopes to the nearby laboratory at the Fossey Fund, which, at this point, was based in Musanze town at the end of each field visit and stored them in the freezer overnight for further analysis the following day. The trichomes remained intact after freezing, and their structure was not altered. In addition, we collected, pressed, and deposited two complete voucher specimens of each study plant species as references for future studies at the Fossey Fund's herbarium.

### 2.3 Sample Analysis

After we removed the samples from the freezer, fresh plant parts of each specimen were mounted together with a scale ruler and photographed using a Crenova camera attached to a UM012C USB Digital Microscope (http://www.crenova.net/) and connected to a computer for further analysis. From each petiole, we photographed the upper and lower surfaces separately (*N* = 135). Each collected leaf (*N* = 135) was divided into three sections (base, middle, and tip), taking reference to where the secondary vein extends from the midvein on both leaf sides. We photographed the intercoastal area on the upper surface and along secondary veins extension on the lower surface. We also took images from each stem segment (*N* = 135 images) and flower stalk (*N* = 45 images). In total, we produced a collection of 1260 images (135 stems, 45 flowers, 270 petioles, 810 leaves) for analysis.

**FIGURE 2:**
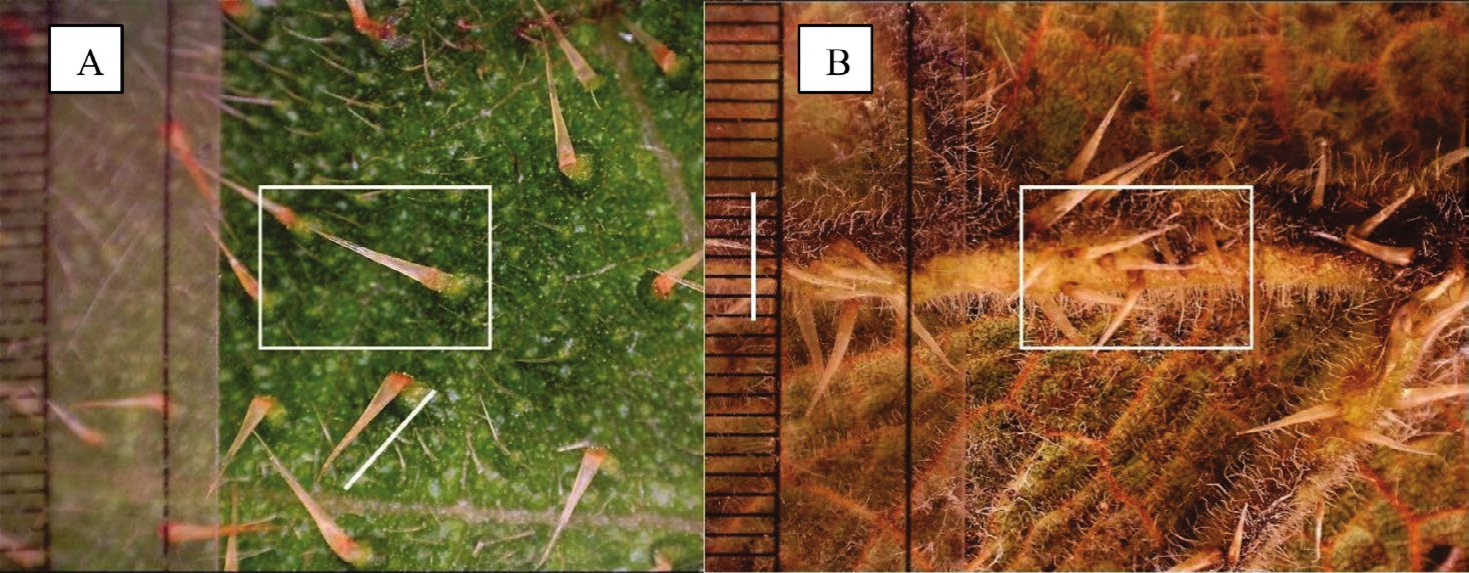
Trichome counts and length measurements; the upper side of leaf (A), the lower side of leaf (B) of *Girardinia bullosa*.

We counted stinging trichomes on each taken image using the scale ruler saved on every image in a 25‐mm^2^ quadrant (Figure 2A) for the upper side of leaves where trichomes were less densely distributed and a 10‐mm^2^ quadrant placed over each image for petioles, stems, flowers, and on lower leaf sides (Figure 2B). Moreover, we selected and measured the length of five flatly laying stinging trichomes and the length of their glandular trichomes base on each image. Using the scale ruler, we measured the length of a line arrow placed along the trichomes (Figure 2A,B).

### 2.4 Feeding Observations on Mountain Gorillas

To provide information about which parts of the stem, which leaves along the stem, and which surface of the leaves of *L. alatipes* were manipulated by gorillas before consumption, we used opportunistic observations on 12 mountain gorillas aged between 6 and 32 years feeding on 29 *Laportea* specimens recorded on video between July 2017 and January 2023. On average, the consumption of two specimens was recorded per gorilla. From those videos, we extracted information about (1) whether gorillas removed leaves located at the upper, middle, or lower stem; (2) which stem sections (upper, middle, and lower) were damaged, consumed, and/or held to support the removal of leaves; and (3) which leaf surface was primarily damaged by gorillas during leaf removal. We also gathered information about the presence/absence of leaves along the stem and the approximate height (in meters) of consumed specimens as an indicator of plant age using average body size estimates for the age of the recorded individually known gorilla (see Galbany et al. 2016) as a reference. The latter information helps to understand whether preferences for leaves at particular stem locations reflect availability.

### 2.5 Data Analysis

We determined the stinging trichome density (trichomes/mm^2^) for each image by dividing the number of counted stinging trichomes per image by the quadrant size (10 or 25 mm^2^). Afterward, we calculated the mean trichome density, mean trichome length, and mean glandular trichome base length for (a) each nettle species studied (including all images from each organ and each specimen), (b) each organ by nettle species, (c) each leaf surface (upper, lower) of *Laportea alatipes*, (d) each leaf location (bottom, middle, top) of *L. alatipes*, and (e) each stem location (bottom, middle, top) for *L. alatipes*.

ANOVA tests were used to compare the means of the three measures (dependent variables), including trichome density, trichome length, and glandular trichome base length, between more than two groups. Where ANOVA revealed a significant difference among groups, we performed Tukey's post hoc tests to determine which groups differed significantly. Dependent *t*‐tests were used to compare the means of the three measures between the two groups. To investigate the link between the importance of each nettle species and their organs studied in the mountain gorilla diet (proportion in their diet) and the nettle species' level of defense through stinging trichomes, we tested for differences in the three measures between nettle species across all organs and between nettle species by organ. From extracted video data, we calculated the proportions of plants: (1) from which gorillas removed leaves located at the upper, middle, and/or lower stem, (2) with the upper, middle, and/or lower stem sections damaged, consumed, and/or held to support the removal of leaves by gorillas, and (3) with primary damage on the upper or lower leaf surface during leaf removal by gorillas. All statistical analyses were performed using R software version 4.4.2.

To compare two proportions, we ran chi‐square tests as recommended by Campbell (2007) using the “prop.test” R function and adjusted the *p* value using the Bonferroni correction (Abdi 2007), which divides the critical *p* value (*α* = 0.05) by the number of dependent tests.

## 3 Results

### 3.1 Trichome Density, Length, and Glandular Trichome Base Length: Between Nettle Species Comparison

**FIGURE 3:**
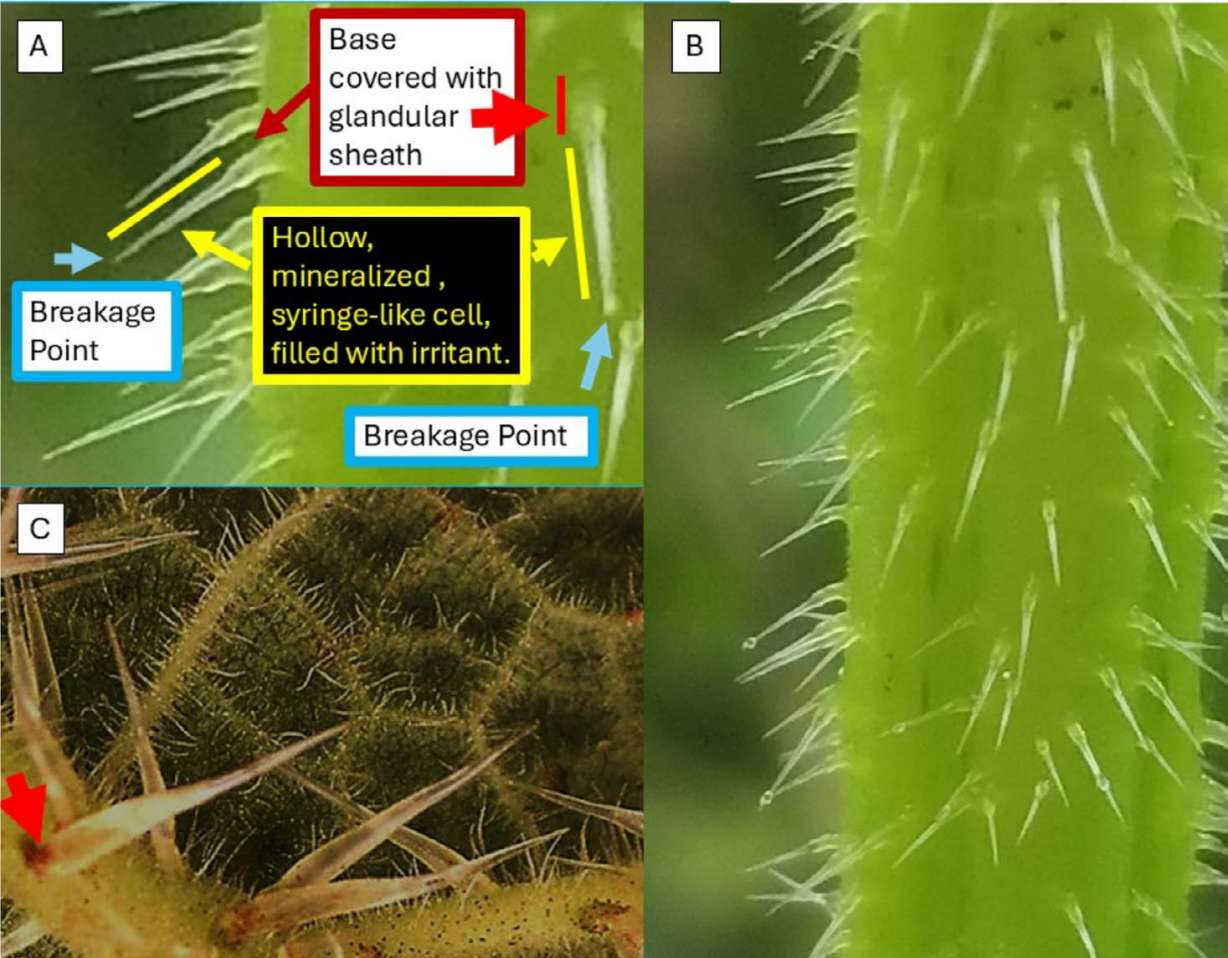
Trichome morphology of the three nettle species; *Urtica massaica* (A, B); arrow indicates long, glandular base. *Girardinia bullosa* (C); arrow indicates shorter base and glandular sheath (appears as a light brown discoloration near trichome base). *Laportea alatipes* (not figured) has the same kind of trichomes as *Girardinia*. Glands surrounding the base of trichomes (red) produce irritants stored in hollow, mineralized, syringe‐like cells (yellow) with tips that break off (blue) and inject irritants into the herbivore.

The trichome morphology of all three nettle species could be distinguished both with the naked eye and under the microscope (Figure 3A–C). The trichome of *Urtica* has a glandular trichome base formed like a stalk and a mineralized tip (Figure 3A). In contrast, *Girardinia* and *Laportea* have a sheathing glandular trichome base with a flexible basal portion (Figure 3B,C).

**FIGURE 4:**
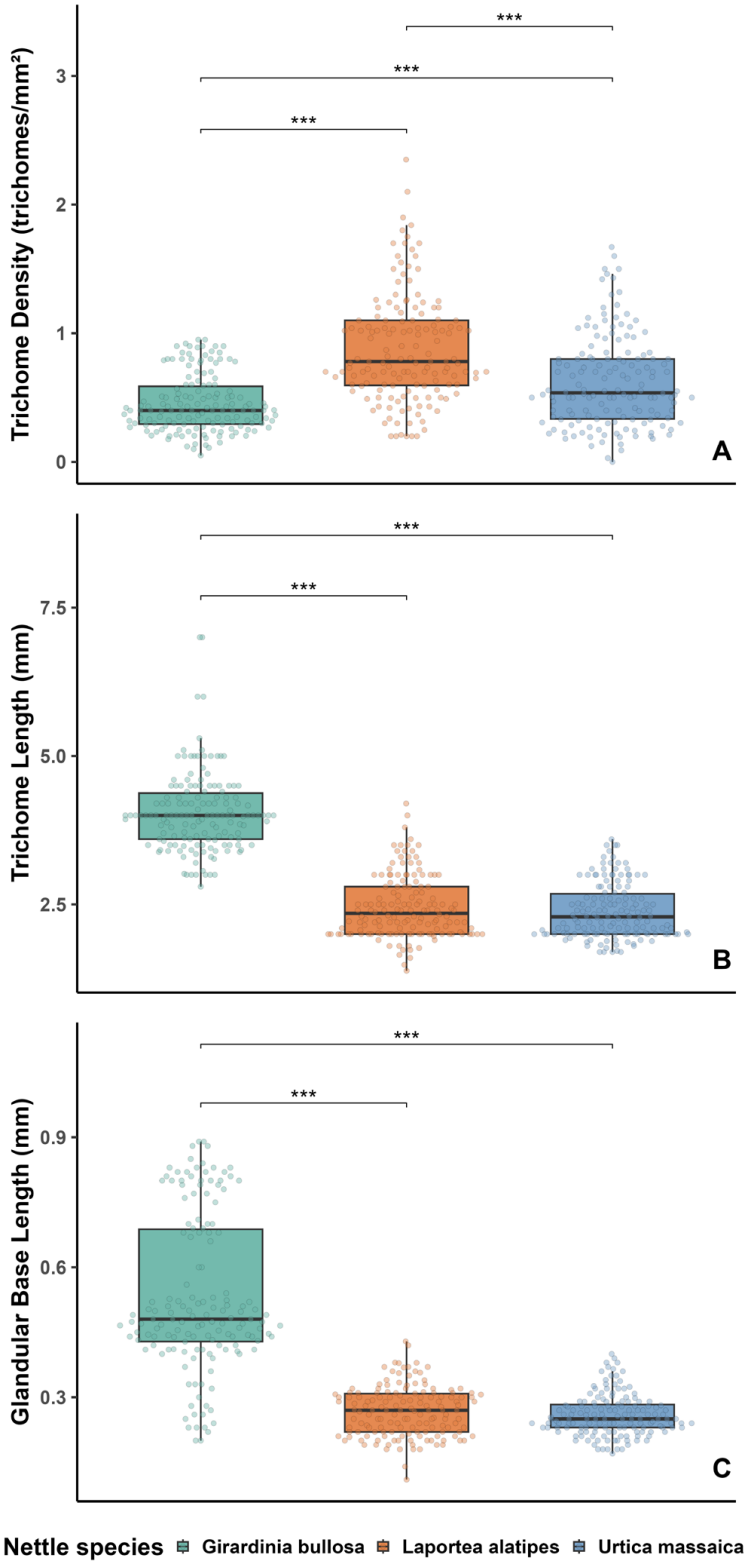
Boxplot showing the median, interquartile range, and raw data points of trichome density (trichomes/mm^2^), trichome length (mm), and glandular trichome base length (mm) of the three study nettle species sampled from the Volcanoes National Park, Rwanda. Brackets and asterisks indicate Tukey post hoc species‐pairwise comparison test results, including trends and significant adjusted *p* values: “.” 0.1 to > 0.05, “*” 0.05 to < 0.01, “**” 0.01 to > 0.001, “***” < 0.001.

The overall mean stinging trichome density differed significantly between the three study nettle species (*F*(2, 447) = 58.141, *p* < 0.001), with the highest density found in *Laportea* and the lowest density found in *Girardinia* (Table S1, Figure 4). Tukey's post hoc tests detected that all species' trichome densities differed significantly from each other (Figure 4). The mean stinging trichome length and mean glandular trichome base length also differed significantly between the three nettle species (trichome length: *F*(2, 447) = 420.785, *p* < 0.001; glandular trichome base length: *F*(2, 447) = 288.895, *p* < 0.001) (Figure 4). *Girardinia*'s trichome length and glandular trichome base length were significantly larger than those of *Laportea* and *Urtica* (Table S1). However, there were no significant differences in the two length measures between *Urtica* and *Laportea*.

### 3.2 Trichome Density, Length, and Glandular Base Length: Between Nettle Species Comparison by Organ

#### 3.2.1 Leaves

**FIGURE 5:**
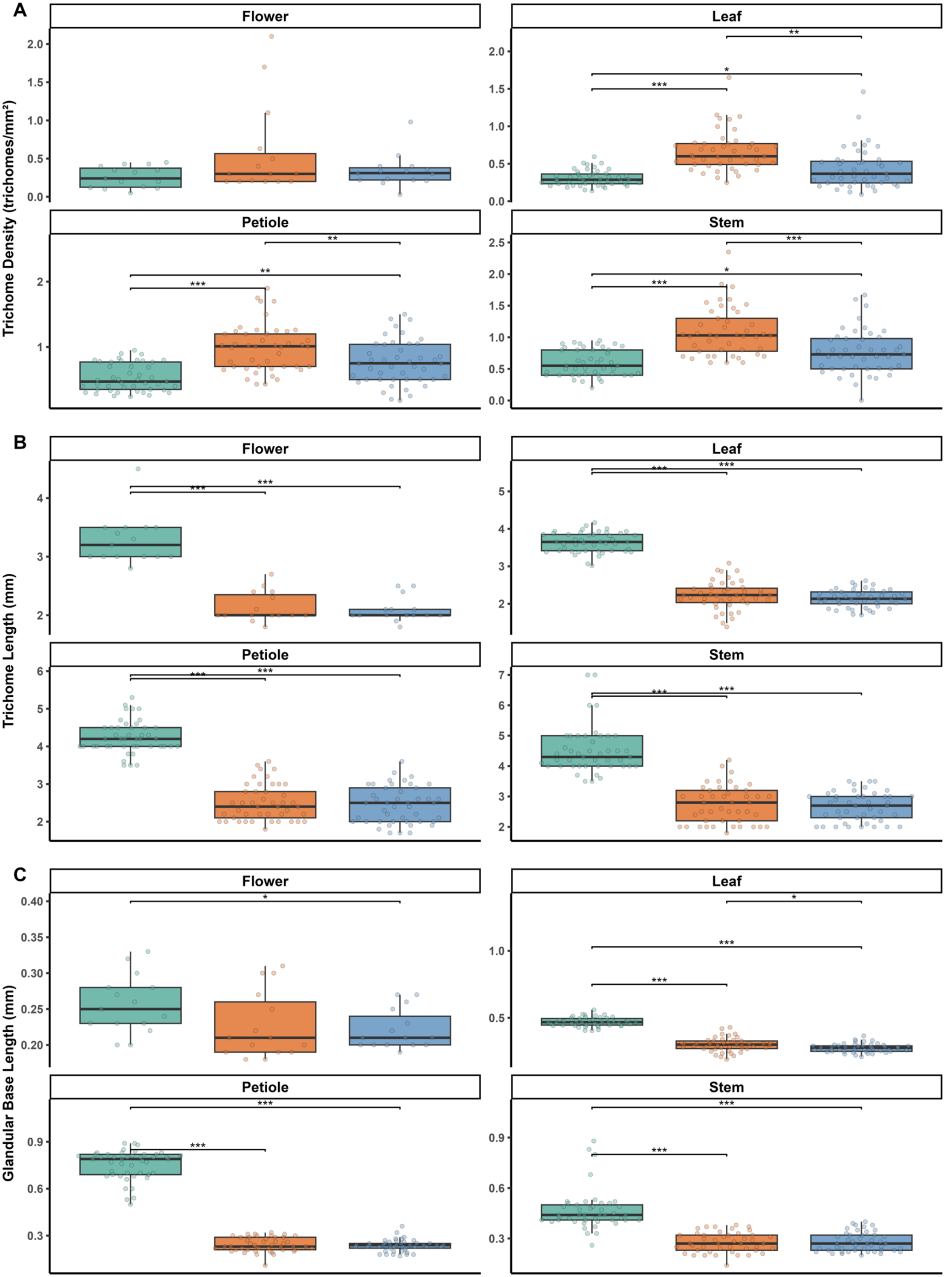
Boxplot showing the median, interquartile range, and raw data points of (A) trichome density (trichomes/mm^2^), (B) trichome length (mm), and (C) glandular trichome base length (mm) by organ and study nettle species sampled from the Volcanoes National Park, Rwanda. Brackets and asterisks indicate Tukey post hoc species‐pairwise comparison test results, including trends and significant adjusted *p* values: “.” 0.1 to > 0.05, “*” 0.05 to < 0.01, “**” 0.01 to > 0.001, “***” < 0.001.

Cross‐species comparison of leaves showed that the mean stinging trichome density, trichome length, and glandular trichome base length differed significantly between the three stinging nettle species (trichome density: *F*(2, 132) = 28.3, *p* < 0.001; trichome length: *F*(2, 132) = 360.7, *p* < 0.001; glandular trichome base length: *F*(2, 132) = 313.8, *p* < 0.001) (Table S2, Figure 5A–C). Pairwise comparison tests showed that *Laportea* had significantly denser trichomes on leaves than *Urtica* and *Girardinia*, whereas *Urtica* leaves had denser trichomes than *Girardinia* leaves (Figure 5). In addition, *Girardinia* had significantly longer trichomes and glandular trichome bases than the other two nettle species.

#### 3.2.2 Petioles

There was a significant nettle species difference in the three mean trichome measures retrieved from petioles (trichome density: *F*(2, 132) = 24.7, *p* < 0.001; trichome length: *F*(2, 132) = 215.9, *p* < 0.001; glandular trichome base length: *F*(2, 132) = 961.4, *p* < 0.001) (Table S2, Figure 5A–C). Post hoc comparisons revealed that *Laportea* had significantly denser trichomes on petioles than *Urtica* and *Girardinia* (Figure 5), whereas trichomes and the glandular trichome bases were longer in *Girardinia* than in *Laportea* and *Urtica*.

#### 3.2.3 Stem Segments

Trichome density and trichome length as well as the glandular trichome base length on stem segments differed significantly among nettle species (density: *F*(2, 132) = 26.5, *p* < 0.001; length: *F*(2, 132) = 120.9, *p* < 0.001; glandular trichome base length: *F*(2, 132) = 77.0, *p* < 0.001) (Table S2, Figure 5A–C). Tukey's post hoc tests showed that *Laportea* had statistically denser trichomes on stems than *Girardinia* and *Urtica* (Figure 5A), whereas *Girardinia* had longer trichomes and glandular trichome bases than both *Laportea* and *Urtica* (Figure 5B,C).

#### 3.2.4 Flowers

Finally, mean trichome density on flowers did not show an interspecific difference (*F*(2, 42) = 2.8, *p* = 0.072) (Table S2, Figure 5A). However, the mean length of trichomes and glandular trichome bases on flowers differed among the study nettle species (trichome length: *F*(2, 42) = 72.4, *p* < 0.001; glandular trichome base length: *F*(2, 42) = 3.43, *p* = 0.043), with *Girardinia* having longer trichomes than *Laportea* and *Urtica*, and longer glandular trichome bases than *Urtica* (Figure 5B,C).

### 3.3 Trichome Density, Trichome Length, and Glandular Trichome Base Length: By Leaf Location on the Stem of *Laportea*

**FIGURE 6:**
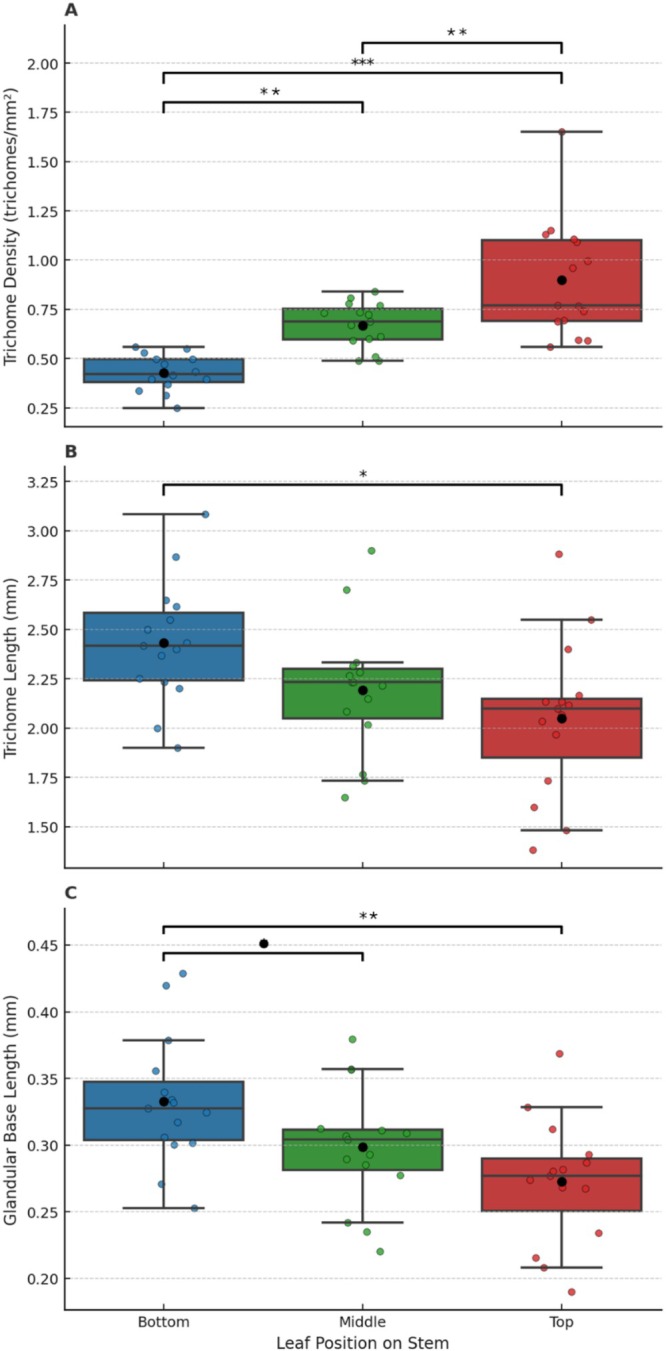
Boxplot showing the median, mean (block dot), interquartile range, and raw data points of trichome density (trichomes/mm^2^), trichome length (mm), and glandular trichome base length (mm) of leaves by location on stems of *Laportea alatipes* species sampled from the Volcanoes National Park, Rwanda. Brackets and asterisks indicate Tukey post hoc pairwise comparison test results, including trends and significant adjusted *p* values: “.” 0.1 to > 0.05, “*” 0.05 to < 0.01, “**” 0.01 to > 0.001, “***” < 0.001.

The mean trichome density increased from bottom leaves to newly emerging leaves (*F*(2, 42) = 22.89, *p* < 0.001) (Table S3, Figure 6). The mean length of trichomes and glandular trichome bases decreased from the bottom leaves to the top leaves (trichome length: *F*(2, 42) = 4.642, *p* = 0.015; glandular trichome base length: *F*(2, 42) = 6.22, *p* = 0.004) (Table S3, Figure 6).

### 3.4 Trichome Density, Trichome Length, and Glandular Trichome Base Length: By Leaf Surface of *Laportae*

**FIGURE 7:**
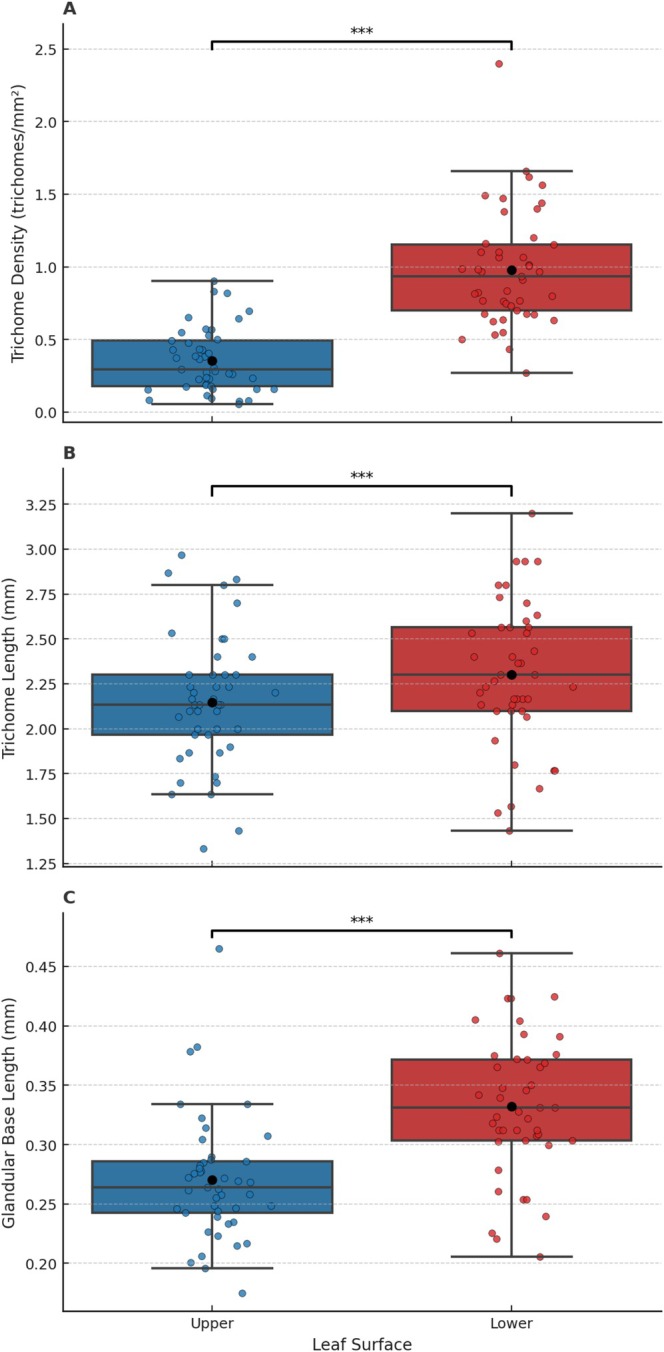
Boxplot showing the median, interquartile range, and raw data points of trichome density (trichomes/mm^2^), trichome length (mm), and glandular trichome base length (mm) by leaf surface of *Laportea alatipes* sampled from the Volcanoes National Park, Rwanda. Brackets and asterisks indicate trends and significant *p* values: “.” 0.1 to > 0.05, “*” 0.05 to < 0.01, “**” 0.01 to > 0.001, “***” < 0.001.

The stinging trichome density was higher on the lower leaf surface than on the upper leaf surface (paired *t*‐test: *t* = −12.11, df = 44, *p* < 0.001) (Table S4, Figure 7). Similarly, the length of trichomes and the glandular trichome bases was larger on the lower leaf surface than on the upper surface (trichome length: *t* = −11.22, df = 44, *p* < 0.011; glandular trichome base length: *t* = −7.21, df = 44, *p* < 0.001) (Table S4, Figure 7).

### 3.5 Trichome Density, Trichome Length, Glandular Trichome Base Length, and Relative Length of Glandular Trichome Base to Total Trichome Length: By Stem Section of *Laportea*

**FIGURE 8:**
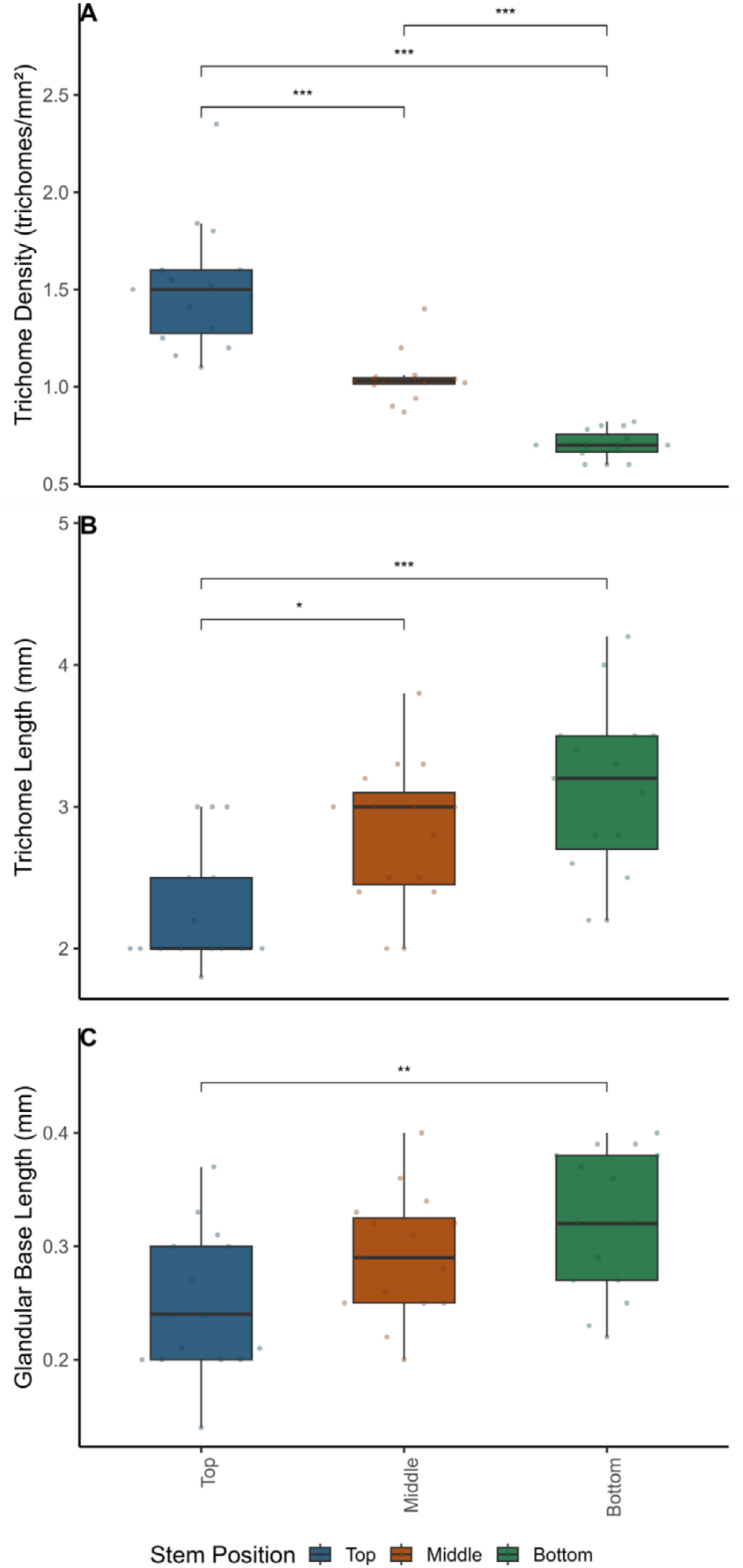
Boxplot showing the median, interquartile range, and raw data points of trichome density (trichomes/mm^2^), trichome length (mm), and glandular trichome base length (mm) by stem section of *Laportea* species sampled from the Volcanoes National Park, Rwanda. Brackets and asterisks indicate Tukey post hoc pairwise comparison test results, including trends and significant *p* values: “.” 0.1 to > 0.05, “*” 0.05 to < 0.01, “**” 0.01 to > 0.001, “***” < 0.001.

The mean trichome density significantly differed by stem section (*F*(1, 42) = 59.97, *p* < 0.001), with a higher density on the top segments of stems compared to the middle and bottom stem segments (Table S5, Figure 8). Trichome length and glandular trichome base length also differed among stem sections (trichome length: *F*(2, 42) = 0.516, *p* = 0.598; glandular trichome base length: *F*(2, 42) = 0.349, *p* = 0.706), indicating a reversed direction with length declining from the bottom to the top stem section (Table S5, Figure 8).

### 3.6 Feeding Techniques When Consuming *Laportea alatipes*

**FIGURE 9:**
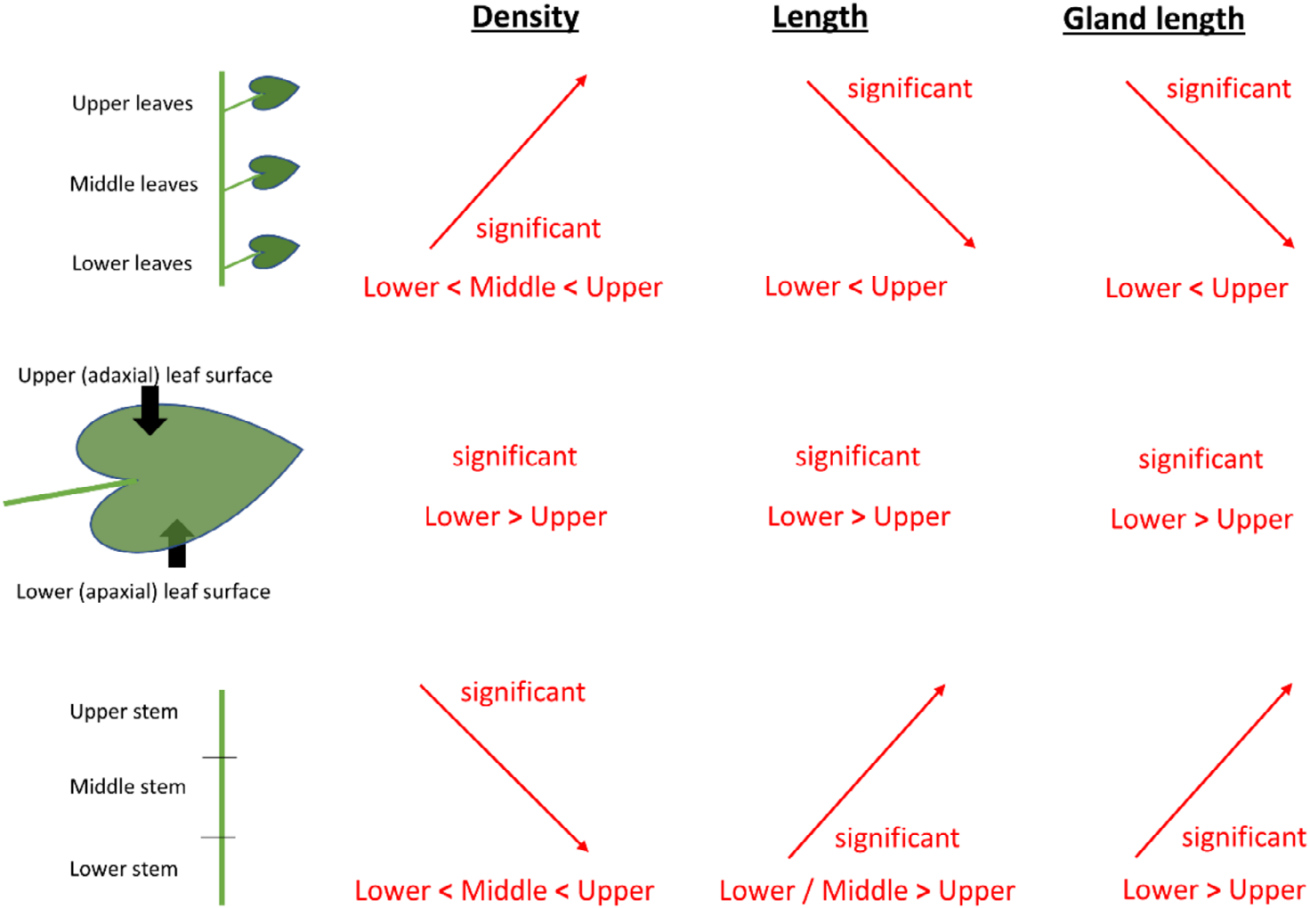
Summary of findings on differences in trichome density, trichome length, and glandular trichome base length on *Laportea alatipes* leaves by surface and leaves and stems by their location.

To test whether described differences in trichome density and length as well as in glandular trichome base length of leaves and stems of *L. alatipes* (summary see Figure 9) reflect adaptive defense strategies in response to predation by mountain gorillas, we analyzed which parts of *L. alatipes* are most frequently manipulated and damaged in the process of gathering and harvesting using video records. In 96.6% of the 29 observations of gorillas consuming *L. alatipes*, they fed on leaves from the upper stem section. Leaves from middle stem sections were targeted in 37.9% of cases, and leaves from the lower stem section were not consumed. Upper leaves were significantly more often consumed by gorillas than middle and lower leaves combined (*χ* ^2^ = 18.125, df = 1, *p* < 0.001, *N* = 29).

Because this finding may be at least in part driven by the presence/absence of leaves at the three stem sections of the consumed specimens, we also investigated feeding preference of leaves by stem sections while controlling for their presence/absence by adjusting expected frequencies for each category (upper, middle, lower). The presence of leaves on all stem sections could be inspected on videos in 22 out of the 29 consumed specimens. Only one of the 22 specimens had leaves on the lower stem section (4.5%), whereas all of them had leaves at the upper stem section, and 13 (59.1%) had leaves at the middle stem section. When upper leaves were present, gorillas consumed them in all cases, while in only 61.5% of the cases, middle leaves were consumed when available, which was a significant difference (*χ* ^2^ = 6.981, df = 1, *p* = 0.008). Finally, the average height of plant specimens harvested by gorillas was 1.2 m (SD ±0.71 m) (range: 0.4–4 m), which is similar to the approximate height of specimens collected in this study (~1.3 m).

During leaf removal, in all observed feeding cases, gorillas predominantly had contact with the lower leaf surface when stripping and folding leaves from the stem, and in only 18.2% of the cases, gorillas would touch the upper and lower leaf surfaces about evenly when they grabbed/picked single leaves to pull them from the stem. This difference was statistically significant (*χ* ^2^ = 40.495, *p* < 0.001, *N* = 29).

Gorillas damaged and/or consumed upper stem sections in 96.6% of observations, which is significantly more frequent than they damaged and/or consumed middle stem sections (20.75%) (*χ* ^2^ = 31.346, *p* < 0.001, *N* = 29). In only three cases (10.3%), gorillas damaged and/or consumed the lower stem section, which is significantly less compared to the upper stem section (*χ* ^2^ = 39.914, *p* < 0.001, *N* = 29) but indifferent to lower stem sections (*χ* ^2^ = 0.526, *p* = 0.463, *N* = 29).

## 4 Discussion

Our results showed that *L. alatipes*, the most commonly consumed of the three studied nettles, had significantly higher stinging trichome density than *U. massaica* and *G. bullosa*. This study evidenced that on *L. alatipes*, the region manipulated or touched most frequently by gorillas develops highest trichome densities. Based on the results of this study, changes in other parameters, such as the length of the trichome or its glandular trichome base, were not associated with herbivory. The multiple roles of trichomes in contributing to fitness in plants are documented (e.g., Pérez‐Estrada et al. 2000). Other roles, such as the ability to reflect light or deter water loss, are potentially influencing the variation of trichome length in *L. alatipes*. The mineralized and reflective nature of trichomes could be contributing to light reflection in lower, shaded regions of the plant body. Our results, suggesting an increase in stinging trichome density with herbivory, are congruent with the findings of other studies focusing on the interactions between mammalian herbivores and mechanical plant defenses (prickles, spines, thorns, and trichomes) (Hurley 2000; Kato et al. 2008; Shikata et al. 2013; Iwamoto et al. 2014; Barton 2016; Charles‐Dominique et al. 2016). In fact, the induction of these physical defense traits is quite widespread, and the ecological and evolutionary effects of these responses, largely density increases, are still relatively unknown. There are numerous environmental, genetic, and physiological interactions responsible for trichome formation (Mustafa et al. 2018; Wang et al. 2021). Herbivory is clearly a stimulus for the formation of physical defense traits that are linked to the fitness of flowering plants (Barton 2016).

### 4.1 Morphology: How Does the Trichome Interact With the Epidermis of the Gorilla

There is some variation at the generic level in the anatomy and morphology of stinging trichomes in the Urticaceae (Urticeae) (Ensikat et al. 2021). Trichomes of *L. alatipes* and *G. bullosa* are structurally the same, although they differ in size and are distinct from those of *U. massaica* (Figure 2). All three species examined in this study, like other members of the Uriceae tribe, have three distinct zones of stinging hairs that could be identified based on their mineralization: the tip, the shafts, and the trichome base (Mustafa et al. 2018). Silica, calcium carbonate, and calcium phosphate are the major compounds identified in biomineralization (Mustafa et al. 2018; Weigend et al. 2018). The siliceous tip interacts with the epidermis of mammals (Mustafa et al. 2018) and may either break off and impinge into the skin for *Urtica* or remain unbroken for *Laportea*. The breakage of the tip and the embedding of its sharp, broken edges into the herbivore's skin could be considered a mechanical adaptation (Ensikat et al. 2021). The combination of impingement and chemical secretion irritates the epidermis, causing some herbivores to immediately avoid further stinging nettle consumption (Fu et al. 2006). We found that *Girardinia* and *Laportea* have a sheathing glandular trichome base that we suggest functions in preventing the trichomes from breaking off but instead allowing them to bend. Despite having similar sheathing structures, our findings revealed that *Girardinia* and *Laportea*'s trichome foot cell and glandular trichome base lengths, as well as total trichome length, are noticeably different and longer on the former. Herbivores, in fact, struggle to harvest, process, and digest large trichomes like those present in *Girardinia*. This provides a plausible explanation for why mountain gorillas are uncommon and unlikely to be found harvesting *G. bullosa* aboveground parts. One may be surprised that *Girardinia*, with trichomes approximately twice the length of the other two species, is not consumed by gorillas, yet golden monkeys, *Cercopithecus mitis kandti* , a sympatric species of the study gorillas, do (personal observation here). We therefore are hesitant to make any assumptions regarding trichome length. Interestingly, a VNP gorilla group ranging in the eastern part of the park was spotted feeding on roots and the pith of *Girardinia* after peeling off the stem's bark from down up, starting from roots (W. Eckardt, personal observation). Conversely, on *Laportea*, the sheathing structure adaptation reduces trichome fragility and breaks off but allows mountain gorillas to easily bend along the vein without penetrating the skin and fold into the leaves during harvesting and prior to ingestion, thereby minimizing physical contact. In this regard, *Laportea* petiole with numerous trichomes that do not break off was recovered from fecal material remains (Tuyisenge et al. 2020), which provides insight into how leaves and petioles with many trichomes are folded. It is one of the most compelling explanations for the previously unknown link between the morphological adaptations of *Laportea* species, harvesting behavior, and feeding technique on stinging nettles (Byrne and Byrne 1993).

### 4.2 Trichomes Interaction With Gorilla, and Manipulation

The evolution of large herbivores has been linked to an increase in mechanical defenses in plant species, including prickles, thorns, trichomes, and the combination of mechanical and chemical defenses in stinging trichomes (Hanley et al. 2007; Charles‐Dominique et al. 2016; Khadgi and Weber 2020). In Urticaceae, our findings further support that stinging nettles increase trichome density to deter mammals from browsing. Other studies focused on structures other than trichomes revealed that prickles have evolved as a common anti‐herbivory defense against mechanical injury (Coverdale et al. 2019). The mechanical defense deters large mammals that damage the plant's structure (Hanley et al. 2007; Iwamoto et al. 2014), whereas the chemical defense prevents small insects that can transmit pathogens or reduce the plant's fitness (Tuberville et al. 1996). Because mechanical defense may necessitate a significant investment in terms of resource allocation (Mutikainen and Walls 1995; Steppuhn and Baldwin 2008), having a combination of defense mechanisms helps stinging nettle adapt to various ecological conditions and increase its chances of survival and reproduction (Hanley et al. 2007; Carmona et al. 2011). Moreover, to ward off herbivores, glandular trichomes in nettles secrete toxic chemicals, such as formic acid, acetylcholine, histamine, serotonin, leukotrienes, and oxalic and tartaric acids (Fu et al. 2006, 2007; Schuurink and Tissier 2020). The amount of secretion is most likely determined by the plant species, the extent of the damage, glandular base length, and maturity of the trichomes. The present study in VNP is consistent with other studies conducted in different geographic areas on Japanese nettles (*Urtica thunbergiana*) and European nettles ( *U. dioica* ), which produce increased stinging hairs on their leaves and stems as defensive strategies (Pullin and Gilbert 1989; Kato et al. 2008). Strategically, as the level of browsing to the plant increases, so does the induced defense (Kohyama et al. 2017). Longer trichomes certainly generate a more powerful mechanical and chemical defense. This is exemplified by the fact that *G. bullosa* had about double the length of trichomes as the other two species and was never seen being touched by gorillas in the VNP study area. Therefore, we propose that this could be an indication that gorillas have certain trichome length limits. Some organs in other species, like *G. bullosa*, are discernible, such as the petiole and bottom part, either leaves or stems, which could be ascribed to longer trichomes and glandular base length discomfort. Moreover, stems, petioles, and leaves of stinging nettle differed in our study in that the stem and petioles have the highest density of trichomes and longest trichomes, followed by the leaves and then the flowers. Mountain gorillas avoid stems but occasionally eat top stem sections, discard petioles but feed on young leaves. It is worth noting that in the case of *Laportea*, gorillas consume the flowers as it is almost unavoidable when stripping off leaves (video analysis) but they are embedded in leaves when ingested due to described feeding techniques (Byrne and Byrne 1993).

We often think of animals as being influenced by their feeding habits. An effective defense, one that deters large herbivores, adds to the fitness of nettles (Kareiva 1999). Species that normally feed on a plant and induce its defense would suffer from the consequences of their previous feeding. Even though plants have defenses, herbivores do not starve, but the less defended plants are frequently relied on for food, whereas other herbivores have adapted to overcome such defenses, as stated in the coevolutionary arms race theory (Kareiva 1999; Ensikat et al. 2021). It is fascinating how mountain gorillas became acquainted with stinging nettle responses and adapted harvesting skills irrespective of the sting and eventually use them as a key food (Watts 1984). Notably, gorillas have larger and stronger bones and muscles in their hands, which allow them to grip and manipulate objects with great force (Deckers et al. 2022; Ruff et al. 2022). Second, gorillas have thicker and tougher skin on their hands compared to other large herbivores. Herbivores' behavior and morphology may contribute to their ability to overcome plant defenses. This distinctive defensive strategy strikingly holds true for stinging nettles growing in VNP under the paramount pressure of large mammals, but mountain gorillas are skilled in handling them and seem insensitive to the stinging nettles, excluding *G. bullosa*, and outdid other herbivores to consume nettles (Plumptre 1991; Byrne et al. 2011; Tennie et al. 2008). Despite their defensive ability, nettle species, as previously discussed, have long been known to have nutritional value and therapeutic properties, making them a staple food source for herbivores (Grauso et al. 2020).

The findings revealed that the trichome density of *L. alatipes* was lower on the bottom leaves and bottom sections of stems where gorillas do not come in contact while stripping leaves off the stem compared to the top leaves and stem sections that are often touched and held while feeding on stinging nettle. Gorillas may devour the top leaves and stem of the nettle plant and remove the leaves of the stem upward. It posits a unique, adapted mechanical feeding technique to damage and lend effortlessly the dense trichomes on newly emerging leaves and the top stem, as well as the process of leaf folding, thereby minimizing physical contact with trichomes. It suggests an immediate counteradaptation of gorillas to respond to the leaf's lower side defense.

Comparing the leaf surface adds value to our understanding of such interactions. Like Pullin and Gilbert (1989), we also found higher trichome density on lower leaf surfaces of *Laportea* compared to upper leaf surfaces, which may explain the nettle species' counteradaptation. It is not surprising, given that gorilla feeding techniques harvest and fold leaves from the bottom upward direction, resulting in larger damage to trichomes on the lower leaf surface. From the vantage point of plant responses, the formation and development of plant trichomes are controlled and coordinated by factors, such as the environment, hormones, regulatory genes, and noncoding RNA (Wang et al. 2021). The present study attempts to elucidate the nature of the connection between stinging trichome densities and positions and the behavioral responses of a large, wild herbivore.

### 4.3 Future Studies

In preparing this contribution, we were surprised to find few recent studies investigating the coevolution and short‐term responses of herbivores and mechanical defenses in plants. There are several aspects of these relationships that are worth exploring.

A relevant future study to this contribution would be examining trichome densities outside the VNP where large herbivores are absent or their ranging patterns can be controlled. Lower trichome density in nettles outside the VNP compared to our study area inside the park could lend further support to our findings.

As stated earlier, stinging trichomes are highly specialized features. However, there are other ways they could be impacting the fitness of the plant, including reducing water loss or reflecting light. Investigations addressing these questions of all three nettle species could be pursued by a series of experiments on water and light exposure using forest restoration areas near the VNP, such as the Ellen Campus of the Dian Fossey Gorilla Fund.

To better understand the benefits for mountain gorillas of feeding on nettles despite their defense mechanisms, future research should focus on the nutritional and medical values of the three stinging study nettles compared to other gorilla food plants and available plants that are not consumed by gorillas. Complementarily, long‐term nettle feeding and health records from gorillas may shed more light on whether gorillas select nettles for their medicinal properties.

One future avenue of research is the inferred connections between the mineralization of trichomes and cystolith formation within the leaves of Urticeae. *Girardinia*, *Laportea*, and *Urtica* are all included in this tribe based on the presence of a combination of stinging hairs and pistillate flowers with four tepals (Friis 1989, 1993). The cystoliths described within this tribe include both punctate and bacilliform forms in *Laportea* and *Urtica* (Miller 1971). Biomineralization is a key process in both trichome and cystolith formation, and assembling both structures requires the excretion of calcium carbonate by the plant (Weigend et al. 2018). The elements involved in the formation of the bases and shafts of Urticeae stinging trichomes include calcium, phosphorus, and silica, while silica constitutes the breakable tip of the stinging cell (Mustafa et al. 2018). Cystoliths have been linked with the following: (1) regulation, sequestration, or excretion of calcium ions and/or ion balance maintenance; (2) the release of CO_2_ in parallel with water molecules; (3) modulation of the light environment; and finally, (4) deterrence to insect herbivory (Karabourniotis et al. 2020). The role of stinging trichomes in deterring mammalian herbivores is overwhelmingly supported by numerous studies, including the present study (see summary in Mustafa et al. 2018). What is unknown at this time is the balance between allocating calcium, phosphorus, and silica for stinging cell formation in younger leaves and the allocation of these elements for cystolith formation in older leaves (Choopan and Grote 2015; Ensikat et al. 2021). It is intriguing to see the decrease in stinging trichome production in older leaves and raises the question of whether there is a balance between investing elemental resources for trichomes in younger leaves and cystoliths in older leaves. Stinging trichomes are costly for the plant to produce and are the primary defense of meristems, reproductive organs, and young leaves (Puustinen et al. 2004; Mymko and Avila‐Sakar 2019). The balancing of the roles cystoliths play in herbivory and physiological plant functions is an intriguing topic worthy of further study. In the case of the present study, we did not build in a component to determine if the gorillas avoided consuming the lower leaves. Future studies should address whether increased mineralization, in the form of cystoliths in older leaves, possibly makes the nutritional value of these leaves lower than the young developing leaves operating as a sink for nutrients. The cost of manipulating and processing younger leaves, with numerous stinging trichomes, is paid off by their higher nutritional quality.

## Author Contributions

**Alphonse Nyandwi:** conceptualization (equal), data curation (equal), formal analysis (equal), investigation (lead), methodology (equal), resources (supporting), software (supporting), supervision (supporting), validation (lead), writing – original draft (lead). **Winnie Eckardt:** data curation (equal), formal analysis (equal), funding acquisition (equal), investigation (equal), methodology (equal), project administration (equal), resources (equal), supervision (equal), validation (equal), visualization (equal), writing – original draft (equal). **Elias Bizuru:** data curation (equal), investigation (equal), project administration (equal), resources (equal), software (equal), supervision (equal), validation (equal), writing – original draft (supporting). **Myriam Mujawamariya:** formal analysis (supporting), investigation (equal), project administration (equal), resources (equal), supervision (equal), writing – original draft (equal). **Melanie L. DeVore:** conceptualization (equal), investigation (equal), methodology (equal), project administration (equal), supervision (equal), visualization (equal), writing – original draft (equal).

## Conflicts of Interest

The authors declare no conflicts of interest.

## Supporting information


Tables S1–S5.

## Data Availability

The data we used for the research analysis involve biological samples (plant specimens). The government of Rwanda through the Rwanda Development Board (RDB) requires that publications resulting from biological samples collected within Rwanda need to be first approved by RDB before submission/publication. In addition, biological samples used for a specific research project approved by RDB cannot be used for other research projects before approval by RDB. Due to these regulations, the datasets cannot be made publicly available on a depository platform but can be requested through the Dian Fossey Gorilla Fund and shared upon approval by the Rwanda Development Board.

## References

[ece371765-bib-0001] Abdi, H. 2007. “The Bonferonni and Šidák Corrections for Multiple Comparisons.” In Encyclopedia of Measurement and Statistics. Sage.

[ece371765-bib-0002] Ågren, J. , and D. W. Schemske . 1993. “The Cost of Defense Against Herbivores: An Experimental Study of Trichome Production in *Brassica rapa* .” American Naturalist 141, no. 2: 338–350. 10.1086/285477 19426086

[ece371765-bib-0003] Baraza, E. , R. Zamora , J. A. Hódar , and J. M. Gómez . 2007. “Plant–Herbivore Interaction: Beyond a Binary Vision.” In Functional Plant Ecology, 481–514. CRC Press.

[ece371765-bib-0004] Barton, K. E. 2016. “Tougher and Thornier: General Patterns in the Induction of Physical Defence Traits.” Functional Ecology 30, no. 2: 181–187.

[ece371765-bib-0005] Burkepile, D. E. , and J. D. Parker . 2017. “Recent Advances in Plant–Herbivore Interactions.” F1000Research 6: 119. 28232868 10.12688/f1000research.10313.1 PMC5302155

[ece371765-bib-0006] Byrne, R. W. 1999. “Imitation Without Intentionality. Using String Parsing to Copy the Organization of Behaviour.” Animal Cognition 2: 63–72.

[ece371765-bib-0007] Byrne, R. W. , and J. M. Byrne . 1993. “Complex Leaf‐Gathering Skills of Mountain Gorillas (*Gorilla g. beringei*): Variability and Standardization.” American Journal of Primatology 31, no. 4: 241–261. 31936992 10.1002/ajp.1350310402

[ece371765-bib-0008] Byrne, R. W. , C. Hobaiter , and M. Klailova . 2011. “Local Traditions in Gorilla Manual Skill: Evidence for Observational Learning of Behavioral Organization.” Animal Cognition 14: 683–693. 21512796 10.1007/s10071-011-0403-8

[ece371765-bib-0009] Campbell, I. 2007. “Chi‐Squared and Fisher–Irwin Tests of Two‐by‐Two Tables With Small Sample Recommendations.” Statistics in Medicine 26, no. 19: 3661–3675. 17315184 10.1002/sim.2832

[ece371765-bib-0010] Carmona, D. , M. J. Lajeunesse , and M. T. Johnson . 2011. “Plant Traits That Predict Resistance to Herbivores.” Functional Ecology 25, no. 2: 358–367.

[ece371765-bib-0011] Charles‐Dominique, T. , T. J. Davies , G. P. Hempson , et al. 2016. “Spiny Plants, Mammal Browsers, and the Origin of African Savannas.” Proceedings of the National Academy of Sciences of the United States of America 113, no. 38: E5572–E5579. 27601649 10.1073/pnas.1607493113 PMC5035896

[ece371765-bib-0012] Choopan, T. , and P. J. Grote . 2015. “Cystoliths in the Leaves of the Genus *Pseuderanthemum* (Acanthaceae) in Thailand.” NU. International Journal of Science 12, no. 2: 13–20.

[ece371765-bib-0013] Coley, P. D. , and J. A. Barone . 1996. “Herbivory and Plant Defenses in Tropical Forests.” Annual Review of Ecology and Systematics 27, no. 1: 305–335.

[ece371765-bib-0014] Coverdale, T. C. , I. J. McGeary , R. D. O'Connell , et al. 2019. “Strong but Opposing Effects of Associational Resistance and Susceptibility on Defense Phenotype in an African Savanna Plant.” Oikos 128, no. 12: 1772–1782.

[ece371765-bib-0015] Dalin, P. , J. Ågren , C. Björkman , P. Huttunen , and K. Kärkkäinen . 2008. “Leaf Trichome Formation and Plant Resistance to Herbivory.” In Induced Plant Resistance to Herbivory, 89–105. Springer Netherlands.

[ece371765-bib-0016] Deckers, K. , Z. J. Tsegai , M. M. Skinner , A. Zeininger , and T. L. Kivell . 2022. “Ontogenetic Changes to Metacarpal Trabecular Bone Structure in Mountain and Western Lowland Gorillas.” Journal of Anatomy 241, no. 1: 82–100. 35122239 10.1111/joa.13630 PMC9178373

[ece371765-bib-0017] Devkota, H. P. , K. R. Paudel , S. Khanal , et al. 2022. “Stinging Nettle ( *Urtica dioica* L.): Nutritional Composition, Bioactive Compounds, and Food Functional Properties.” Molecules 27: 5219. 10.3390/molecules27165219. 36014458 PMC9413031

[ece371765-bib-0018] DeVore, M. L. , A. Nyandwi , W. Eckardt , E. Bizuru , M. Mujawamariya , and K. B. Pigg . 2020. “Urticaceae Leaves With Stinging Trichomes Were Already Present in Latest Early Eocene Okanogan Highlands, British Columbia, Canada.” American Journal of Botany 107, no. 10: 1449–1456. 33091153 10.1002/ajb2.1548

[ece371765-bib-0019] Dushimirimana, P. 2019. “Diet Overlap Between Mountain Gorillas and Other Sympatric Large Herbivores in Volcanoes National Park (VNP).” Unpublished Bachelor's thesis, University of Rwanda.

[ece371765-bib-0020] Ensikat, H. J. , H. Wessely , M. Engeser , and M. Weigend . 2021. “Distribution, Ecology, Chemistry and Toxicology of Plant Stinging Hairs.” Toxins 13, no. 2: 141. 33668609 10.3390/toxins13020141 PMC7918447

[ece371765-bib-0021] Fordyce, J. A. , and A. A. Agrawal . 2001. “The Role of Plant Trichomes and Caterpillar Group Size on Growth and Defence of the Pipevine Swallowtail *Battus philenor* .” Journal of Animal Ecology 70, no. 6: 997–1005.

[ece371765-bib-0022] Friis, I. 1989. “A Revision of *Pilea* (Urticaceae) in Africa.” Kew Bulletin 44: 557–600.

[ece371765-bib-0023] Friis, I. 1993. “Urticaceae.” In Flowering Plants Dicotyledons. The Families and Genera of Vascular Plants, edited by K. Kubitzki , J. G. Rohwer , and V. Bittrich , vol. 2. Springer. 10.1007/978-3-662-02899-5_76.

[ece371765-bib-0024] Fu, H. Y. , S. J. Chen , R. F. Chen , W. H. Ding , L. L. Kuo‐Huang , and R. N. Huang . 2006. “Identification of Oxalic Acid and Tartaric Acid as Major Persistent Pain‐Inducing Toxins in the Stinging Hairs of the Nettle, *Urtica thunbergiana* .” Annals of Botany 98, no. 1: 57–65. 16675601 10.1093/aob/mcl089 PMC2803540

[ece371765-bib-0025] Fu, H. Y. , S. J. Chen , R. F. Chen , L. L. Kuo‐Huang , and R. N. Huang . 2007. “Why Do Nettles Sting? About Stinging Hairs Looking Simple but Acting Complex.” Functional Plant Science and Biotechnology 1, no. 1: 46–55.

[ece371765-bib-0026] Galbany, J. , O. Imanizabayo , A. Romero , et al. 2016. “Tooth Wear and Feeding Ecology in Mountain Gorillas From Volcanoes National Park, Rwanda.” American Journal of Physical Anthropology 159, no. 3: 457–465. 26597436 10.1002/ajpa.22897

[ece371765-bib-0027] Granjon, A. C. , M. M. Robbins , J. Arinaitwe , et al. 2020. “Estimating Abundance and Growth Rates in a Wild Mountain Gorilla Population.” Animal Conservation 23, no. 4: 455–465.

[ece371765-bib-0028] Grauso, L. , B. de Falco , V. Lanzotti , and R. Motti . 2020. “Stinging Nettle, *Urtica dioica* L.: Botanical, Phytochemical and Pharmacological Overview.” Phytochemistry Reviews 19: 1341–1377.

[ece371765-bib-0029] Gray, M. , A. McNeilage , K. Fawcett , et al. 2010. “Censusing the Mountain Gorillas in the Virunga Volcanoes: Complete Sweep Method Versus Monitoring.” African Journal of Ecology 48, no. 3: 588–599.

[ece371765-bib-0030] Gray, M. , J. Roy , L. Vigilant , et al. 2013. “Genetic Census Reveals Increased but Uneven Growth of a Critically Endangered Mountain Gorilla Population.” Biological Conservation 158: 230–238.

[ece371765-bib-0031] Grueter, C. C. , F. Ndamiyabo , A. J. Plumptre , et al. 2013. “Long‐Term Temporal and Spatial Dynamics of Food Availability for Endangered Mountain Gorillas in Volcanoes National Park, Rwanda.” American Journal of Primatology 75, no. 3: 267–280. 23208819 10.1002/ajp.22102

[ece371765-bib-0032] Hagen, M. , W. D. Kissling , C. Rasmussen , et al. 2012. “Biodiversity, Species Interactions and Ecological Networks in a Fragmented World.” In Advances in Ecological Research, vol. 46, 89–210. Academic Press.

[ece371765-bib-0033] Hanley, M. E. , B. B. Lamont , M. M. Fairbanks , and C. M. Rafferty . 2007. “Plant Structural Traits and Their Role in Anti‐Herbivore Defence.” Perspectives in Plant Ecology, Evolution and Systematics 8, no. 4: 157–178.

[ece371765-bib-0034] Harcourt, A. H. , and K. J. Stewart . 2007. Gorilla Society: Conflict, Compromise, and Cooperation Between Sexes. University of Chicago Press.

[ece371765-bib-0035] Hurley, M. 2000. “Growth Dynamics and Leaf Quality of the Stinging Trees *Dendrocnide moroides* and *Dendrocnide cordifolia* (Family Urticaceae) in Australian Tropical Rainforest: Implications for Herbivores.” Australian Journal of Botany 48, no. 2: 191–201.

[ece371765-bib-0036] Ihimbazwe, H. , J. D. Tuyizere , L. Kayitete , et al. 2025. “Dietary Variability Among Mountain Gorilla Groups Across Volcanoes National Park, Rwanda.” Ecology and Evolution 15: e71192. 10.1002/ece3.71192. 40386491 PMC12081832

[ece371765-bib-0037] Iwamoto, M. , C. Horikawa , M. Shikata , N. Wasaka , T. Kato , and H. Sato . 2014. “Stinging Hairs on the Japanese Nettle *Urtica thunbergiana* Have a Defensive Function Against Mammalian but Not Insect Herbivores.” Ecological Research 29: 455–462.

[ece371765-bib-0038] Jordano, P. 2016. “Chasing Ecological Interactions.” PLoS Biology 14, no. 9: e1002559. 27631692 10.1371/journal.pbio.1002559 PMC5025190

[ece371765-bib-0039] Kalpers, J. , E. A. Williamson , M. M. Robbins , et al. 2003. “Gorillas in the Crossfire: Population Dynamics of the Virunga Mountain Gorillas Over the Past Three Decades.” Oryx 37, no. 3: 326–337.

[ece371765-bib-0040] Karabourniotis, G. , G. Liakopoulos , D. Nikolopoulos , and P. Bresta . 2020. “Protective and Defensive Roles of Non‐Glandular Trichomes Against Multiple Stresses: Structure–Function Coordination.” Journal of Forestry Research 31, no. 1: 1–12.

[ece371765-bib-0041] Kareiva, P. 1999. “Coevolutionary Arms Races: Is Victory Possible?” Proceedings of the National Academy of Sciences of the United States of America 96, no. 1: 8–10. 9874761 10.1073/pnas.96.1.8 PMC33539

[ece371765-bib-0042] Karger, D. N. , O. Conrad , J. Böhner , et al. 2017. “Climatologies at High Resolution for the Earth's Land Surface Areas.” Scientific Data 4, no. 1: 1–20. 10.1038/sdata.2017.122 PMC5584396 28872642

[ece371765-bib-0043] Kato, T. , K. Ishida , and H. Sato . 2008. “The Evolution of Nettle Resistance to Heavy Deer Browsing.” Ecological Research 23: 339–345.

[ece371765-bib-0044] Khadgi, A. , and C. A. Weber . 2020. “Morphological Characterization of Prickled and Prickle‐Free *Rubus* Using Scanning Electron Microscopy.” HortScience 55, no. 5: 676–683.

[ece371765-bib-0045] Kohyama, T. , C. Horikawa , S. Kawai , M. Shikata , T. Kato , and H. Sato . 2017. “Differential Butterfly Performance on Host Plant Variants From Populations Under Intense vs. Low Mammalian Herbivory.” Ecosphere 8, no. 1: e01568.

[ece371765-bib-0046] Mahlangeni, N. T. , R. Moodley , and S. B. Jonnalagadda . 2020. “Nutritional Value, Antioxidant and Antidiabetic Properties of Nettles (*Laportea alatipes* and *Obetia tenax*).” Scientific Reports 10: 9762. 10.1038/s41598-020-67055-w. 32555290 PMC7300021

[ece371765-bib-0047] McNeilage, A. 2001. “Diet and Habitat Use of Two Mountain Gorilla Groups in Contrasting Habitats in the Virungas.” In Mountain Gorillas Thirty Years of Research at Karisoke, edited by M. M. Robbins , P. Sicotte , and K. J. Stewart , 265–292. Cambridge University Press.

[ece371765-bib-0048] Mehta, H. , and C. Katee . 2005. Virunga Massif Sustainable Tourism Development Plan: DR Congo, Rwanda and Uganda. ECDA.

[ece371765-bib-0049] Miller, N. G. 1971. “The Genera of the Urticaceae in the Southeastern United States.” Journal of the Arnold Arboretum 52, no. 1: 40–68.

[ece371765-bib-0050] Mithöfer, A. , and W. Boland . 2012. “Plant Defense Against Herbivores: Chemical Aspects.” Annual Review of Plant Biology 63: 431–450. 10.1146/annurev-arplant-042110-103854 22404468

[ece371765-bib-0051] Mustafa, A. , H. J. Ensikat , and M. Weigend . 2018. “Stinging Hair Morphology and Wall Biomineralization Across Five Plant Families: Conserved Morphology Versus Divergent Cell Wall Composition.” American Journal of Botany 105, no. 7: 1109–1122. 30080249 10.1002/ajb2.1136

[ece371765-bib-0052] Mutikainen, P. , and M. Walls . 1995. “Growth, Reproduction and Defence in Nettles: Responses to Herbivory Modified by Competition and Fertilization.” Oecologia 104: 487–495. 28307664 10.1007/BF00341346

[ece371765-bib-0053] Mymko, D. , and G. Avila‐Sakar . 2019. “The Influence of Leaf Ontogenetic Stage and Plant Reproductive Phenology on Trichome Density and Constitutive Resistance in Six Tomato Varieties.” Arthropod‐Plant Interactions 13, no. 5: 797–803.

[ece371765-bib-0054] Orrock, J. L. , E. T. Borer , L. A. Brudvig , et al. 2015. “A Continent‐Wide Study Reveals Clear Relationships Between Regional Abiotic Conditions and Post‐Dispersal Seed Predation.” Journal of Biogeography 42, no. 4: 662–670.

[ece371765-bib-0055] Owiunji, I. , D. Nkuutu , D. Kujirakwinja , et al. 2005. “The Biodiversity of the Virunga Volcanoes.” Unpublished Report. Wildlife Conservation Society.

[ece371765-bib-0056] Pérez‐Estrada, L. B. , Z. Cano‐Santana , and K. Oyama . 2000. “Variation in Leaf Trichomes of *Wigandia urens* : Environmental Factors and Physiological Consequences.” Tree Physiology 20, no. 9: 629–632. 12651428 10.1093/treephys/20.9.629

[ece371765-bib-0057] Plumptre, A. J. 1991. “Plant–Herbivore Dynamics in the Birungas.” Doctoral diss., University of Bristol.

[ece371765-bib-0058] Plumptre, A. J. 1996. “Modelling the Impact of Large Herbivores on the Food Supply of Mountain Gorillas and Implications for Management.” Biological Conservation 75, no. 2: 147–155.

[ece371765-bib-0059] Pollard, A. J. , and D. Briggs . 1984. “Genecological Studies of *Urtica dioica* L. III. Stinging Hairs and Plant–Herbivore Interactions.” New Phytologist 97, no. 3: 507–522.

[ece371765-bib-0060] Pullin, A. S. , and J. E. Gilbert . 1989. “The Stinging Nettle, *Urtica dioica* , Increases Trichome Density After Herbivore and Mechanical Damage.” Oikos 54: 275–280.

[ece371765-bib-0061] Puustinen, S. , T. Koskela , and P. Mutikainen . 2004. “Direct and Ecological Costs of Resistance and Tolerance in the Stinging Nettle.” Oecologia 139: 76–82. 14745650 10.1007/s00442-004-1488-4

[ece371765-bib-0062] Ruff, C. B. , J. A. Junno , M. L. Burgess , et al. 2022. “Body Proportions and Environmental Adaptation in Gorillas.” American Journal of Biological Anthropology 177, no. 3: 501–529. 36787793 10.1002/ajpa.24443

[ece371765-bib-0063] Schilmiller, A. L. , R. L. Last , and E. Pichersky . 2008. “Harnessing Plant Trichome Biochemistry for the Production of Useful Compounds.” Plant Journal 54, no. 4: 702–711. 10.1111/j.1365-313X.2008.03432.x 18476873

[ece371765-bib-0064] Schuurink, R. , and A. Tissier . 2020. “Glandular Trichomes: Micro‐Organs With Model Status?” New Phytologist 225, no. 6: 2251–2266. 31651036 10.1111/nph.16283

[ece371765-bib-0065] Shikata, M. , T. Kato , E. Shibata , and H. Sato . 2013. “Among‐Population Variation in Resistance Traits of a Nettle and Its Relationship With Deer Habitat Use Frequency.” Ecological Research 28: 207–216.

[ece371765-bib-0066] Steppuhn, A. , and I. T. Baldwin . 2008. “Induced Defenses and the Cost–Benefit Paradigm.” In Induced Plant Resistance to Herbivory, 61–83. Springer Netherlands.

[ece371765-bib-0067] Tennie, C. , D. Hedwig , J. Call , and M. Tomasello . 2008. “An Experimental Study of Nettle Feeding in Captive Gorillas.” American Journal of Primatology 70, no. 6: 584–593. 18330896 10.1002/ajp.20532

[ece371765-bib-0068] Thurston, E. L. 1969. “An Anatomical and Fine Structure Study of Sting Hairs in Some Members of the Urticaceae, Euphorbiaceae and Loasaceae.” Iowa State University.

[ece371765-bib-0079] Thurston, E. L. , and N. R. Lersten . 1969. “The morphology and toxicology of plant stinging hairs.” The Botanical Review 35, no. 4: 393–412.

[ece371765-bib-0080] Thurston, E. L. , 1974. “Morphology, fine structure, and ontogeny of the stinging emergence of Urtica dioica.” American Journal of Botany 61, no. 8: 809–817.

[ece371765-bib-0069] Traw, B. M. , and T. E. Dawson . 2002. “Differential Induction of Trichomes by Three Herbivores of Black Mustard.” Oecologia 131: 526–532. 28547547 10.1007/s00442-002-0924-6

[ece371765-bib-0070] Tuberville, T. D. , P. G. Dudley , and A. J. Pollard . 1996. “Responses of Invertebrate Herbivores to Stinging Trichomes of *Urtica dioica* and *Laportea canadensis* .” Oikos 75: 83–88.

[ece371765-bib-0071] Tuyisenge, M. F. , W. Eckardt , S. Nshutiyayesu , and M. Devore . 2020. “A Simple and Environmentally Friendly Field Method for Fecal Analysis of Herbivore Diet.” Wildlife Society Bulletin 44, no. 4: 807–817.

[ece371765-bib-0072] Twahirwa, J. C. , D. Tuyisingize , A. Sekabanza , et al. 2023. “Positive Population Trends Among Meso‐ and Megaherbivores Follow Intensive Conservation Efforts in Volcanoes National Park, Rwanda.” Wildlife Biology 2025: e01118.

[ece371765-bib-0073] Vedder, A. L. 1984. “Movement Patterns of a Group of Free‐Ranging Mountain Gorillas ( *Gorilla gorilla* *beringei*) and Their Relation to Food Availability.” American Journal of Primatology 7, no. 2: 73–88. 32131568 10.1002/ajp.1350070202

[ece371765-bib-0074] Wang, X. , C. Shen , P. Meng , G. Tan , and L. Lv . 2021. “Analysis and Review of Trichomes in Plants.” BMC Plant Biology 21: 1–11. 33526015 10.1186/s12870-021-02840-x PMC7852143

[ece371765-bib-0075] War, A. R. , G. K. Taggar , B. Hussain , M. S. Taggar , R. M. Nair , and H. C. Sharma . 2018. “Plant Defence Against Herbivory and Insect Adaptations.” AoB Plants 10, no. 4: ply037.

[ece371765-bib-0076] Watts, D. P. 1984. “Composition and Variability of Mountain Gorilla Diets in the Central Virungas.” American Journal of Primatology 7, no. 4: 323–356. 32106635 10.1002/ajp.1350070403

[ece371765-bib-0077] Weigend, M. , A. Mustafa , and H. J. Ensikat . 2018. “Calcium Phosphate in Plant Trichomes: The Overlooked Biomineral.” Planta 247: 277–285. 29234879 10.1007/s00425-017-2826-1

[ece371765-bib-0078] Yang, L. , K. S. Wen , X. Ruan , Y. X. Zhao , F. Wei , and Q. Wang . 2018. “Response of Plant Secondary Metabolites to Environmental Factors.” Molecules 23, no. 4: 762. 29584636 10.3390/molecules23040762 PMC6017249

