# Supplementary material for: Stinging Trichome Density and Morphology of Three Nettle Species Reflect Mountain Gorillas' Feeding Behavior
Source: Ecol Evol. 2025 Jul 22;15(7):e71765. doi: 10.1002/ece3.71765 (PMC12283244; doi:10.1002/ece3.71765)
Supplement: Supplementary file 1 — Tables S1–S5. [file ECE3-15-e71765-s001.docx]

STINGING TRICHOME DENSITY AND MORPHOLOGY OF THREE NETTLE SPECIES REFLECT MOUNTAIN GORILLAS' FEEDING BEHAVIOR

**Alphonse Nyandwi^1,2^| Winnie Eckardt^2^| Elias Bizuru^1^| Myriam Mujawamariya^1^| Melanie L. DeVore^3^**

**Supplementary materials**

| **Supplementary Table 1**: Mean and standard deviation (±SD) of trichome density (trichomes/mm2), trichome length (mm), and glandular trichome base length (mm) of the three study nettle species sampled from the Volcanoes National Park, Rwanda.   \|  \| Trichome density (trichomes/mm2) \| Trichome length  (mm) \| Glandular base length (mm) \| \| --- \| --- \| --- \| --- \| \| Species \| Mean±SD \| Mean±SD \| Mean±SD \| \| Urtica massaica \| 0.62±0.36 \| 2.39±0.46 \| 0.25±0.047 \| \| Girardinia bullosa \| 0.45±0.22 \| 4.05±0.67 \| 0.53±0.17 \| \| Laportea alatipes \| 0.88±0.42 \| 2.45±0.53 \| 0.26±0.05 \|   **Supplementary Table 2**: Mean and standard deviation (±SD) of trichome density (trichomes/mm2), trichome length (mm), and glandular trichome base length (mm) by organ and study nettle species sampled from the Volcanoes National Park, Rwanda.   \|  \| Trichome density (trichomes/mm2) \| Trichome length  (mm) \| Glandular base length (mm) \| \| --- \| --- \| --- \| --- \| \| Species and organs \| Mean±SD \| Mean±SD \| Mean±SD \| \| Leave \|  \|  \|  \| \| Urtica massaica \| 0.130±0.24 \| 0.640±0.99 \| 0.080±0.13 \| \| Girardinia bullosa \| 0.090±0.15 \| 1.090±1.68 \| 0.140±0.21 \| \| Laportea alatipes \| 0.190±0.33 \| 0.660±1.04 \| 0.090±0.14 \| \| Petioles \|  \|  \|  \| \| Urtica massaica \| 0.220±0.39 \| 0.730±1.15 \| 0.070±0.11 \| \| Girardinia bullosa \| 0.150±0.26 \| 1.270±1.96 \| 0.220±0.34 \| \| Laportea alatipes \| 0.290±0.49 \| 0.750±1.18 \| 0.070±0.11 \| \| Stems \|  \|  \|  \| \| Urtica massaica \| 0.230±0.40 \| 0.800±1.25 \| 0.080±0.13 \| \| Girardinia bullosa \| 0.170±0.29 \| 1.350±2.11 \| 0.140±0.22 \| \| Laportea alatipes \| 0.320±0.54 \| 0.820±1.30 \| 0.080±0.13 \| \| Flowers \|  \|  \|  \| \| Urtica massaica \| 0.029±0.09 \| 0.200±0.63 \| 0.020±0.06 \| \| Girardinia bullosa \| 0.025±0.08 \| 0.320±0.99 \| 0.020±0.07 \| \| Laportea alatipes \| 0.057±0.25 \| 0.210±0.64 \| 0.020±0.07 \|   **Supplementary Table 3**: Mean and standard deviation (±SD) of trichome density (trichomes/mm2), trichome length (mm), and glandular trichome base length (mm) of leaves by location on stems of Laportea alatipes species sampled from the Volcanoes National Park, Rwanda.   \|  \| Trichome density (trichomes/mm2) \| Trichome length  (mm) \| Glandular base length (mm) \| \| --- \| --- \| --- \| --- \| \| Location of leaf \| Mean±SD \| Mean±SD \| Mean±SD \| \| Top \| 0.29±0.46 \| 0.68±1.00 \| 0.09±0.13 \| \| Middle \| 0.22±0.32 \| 0.73±1.06 \| 0.09±0.14 \| \| Bottom \| 0.14±0.21 \| 0.81±1.17 \| 0.11±0.16 \|   **Supplementary Table 4:** Mean and standard deviation (±SD) of trichome density (trichomes/mm2), trichome length (mm), and glandular trichome base length (mm) by leaf surface of Laportea alatipes sampled from the Volcanoes National Park, Rwanda.   \|  \| Trichome density (trichomes/mm2) \| Trichome length  (mm) \| \| Glandular base length (mm) \| \| --- \| --- \| --- \| --- \| --- \| \| Leaf surface \| Mean±SD \| Mean±SD \| \| Mean±SD \| \| Upper side \| 0.35±0.21 \| 2.14±0.36 \| 0.27±0.05 \| \| \| Lower side \| 0.97±0.39 \| 2.30±0.39 \| 0.33±0.05 \| \|   **Supplementary Table 5:** Mean and standard deviation (±SD) of trichome density (trichomes/mm2), trichome length (mm), and glandular trichome base length (mm) by stem section of Laportea alatipes sampled from the Volcanoes National Park, Rwanda.   \|  \| Trichome density (trichomes/mm2) \| Trichome length  (mm) \| Glandular base length (mm) \| \| --- \| --- \| --- \| --- \| \| Location on stem \| Mean±SD \| Mean±SD \| Mean±SD \| \| Top \| 0.50±0.74 \| 0.76±1.11 \| 0.08±0.12 \| \| Middle \| 0.35±0.50 \| 0.94±1.37 \| 0.10±0.14 \| \| Bottom \| 0.23±0.34 \| 1.04±1.53 \| 0.11±0.16 \| |
| --- | --- | --- | --- | --- | --- | --- | --- | --- | --- | --- | --- | --- | --- | --- | --- | --- | --- | --- | --- | --- | --- | --- | --- | --- | --- | --- | --- | --- | --- | --- | --- | --- | --- | --- | --- | --- | --- | --- | --- | --- | --- | --- | --- | --- | --- | --- | --- | --- | --- | --- | --- | --- | --- | --- | --- | --- | --- | --- | --- | --- | --- | --- | --- | --- | --- | --- | --- | --- | --- | --- | --- | --- | --- | --- | --- | --- | --- | --- | --- | --- | --- | --- | --- | --- | --- | --- | --- | --- | --- | --- | --- | --- | --- | --- | --- | --- | --- | --- | --- | --- | --- | --- | --- | --- | --- | --- | --- | --- | --- | --- | --- | --- | --- | --- | --- | --- | --- | --- | --- | --- | --- | --- | --- | --- | --- | --- | --- | --- | --- | --- | --- | --- | --- | --- | --- | --- | --- | --- | --- | --- | --- | --- | --- | --- | --- | --- | --- | --- | --- | --- | --- | --- |
